# GCKIII kinases control hepatocellular lipid homeostasis via shared mode of action

**DOI:** 10.1016/j.jlr.2024.100669

**Published:** 2024-10-10

**Authors:** Emmelie Cansby, Mara Caputo, Emma Andersson, Rasool Saghaleyni, Marcus Henricsson, Ying Xia, Bernice Asiedu, Matthias Blüher, L. Thomas Svensson, Andrew J. Hoy, Margit Mahlapuu

**Affiliations:** 1 Department of Chemistry and Molecular Biology, University of Gothenburg and Sahlgrenska University Hospital, Gothenburg, Sweden; 2 Department of Life Sciences, National Bioinformatics Infrastructure Sweden, Science for Life Laboratory, Chalmers University of Technology, Gothenburg, Sweden; 3 Translational Science and Experimental Medicine, Research and Early Development, Cardiovascular, Renal and Metabolism, BioPharmaceuticals R&D, AstraZeneca, Gothenburg, Sweden; 4 Department of Molecular and Clinical Medicine/Wallenberg Laboratory, Institute of Medicine, University of Gothenburg and Sahlgrenska University Hospital, Gothenburg, Sweden; 5 Helmholtz Institute for Metabolic, Obesity, and Vascular Research (HI-MAG) of the Helmholtz Zentrum München, University of Leipzig and University Hospital Leipzig, Leipzig, Germany; 6 School of Medical Sciences, Charles Perkins Centre, University of Sydney, Sydney, NSW, Australia

**Keywords:** MASLD, MASH, lipid droplets, liver, lipotoxicity, lipidomics, triacylglycerol

## Abstract

Metabolic dysfunction–associated steatotic liver disease has emerged as a leading global cause of chronic liver disease. Our recent translational investigations have shown that the STE20-type kinases comprising the GCKIII subfamily—MST3, STK25, and MST4—associate with hepatic lipid droplets and regulate ectopic fat storage in the liver; however, the mode of action of these proteins remains to be resolved. By comparing different combinations of the silencing of MST3, STK25, and/or MST4 in immortalized human hepatocytes, we found that their single knockdown results in a similar reduction in hepatocellular lipid content and metabolic stress, without any additive or synergistic effects observed when all three kinases are simultaneously depleted. A genome-wide yeast two-hybrid screen of the human hepatocyte library identified several interaction partners contributing to the GCKIII-mediated regulation of liver lipid homeostasis, that is, PDCD10 that protects MST3, STK25, and MST4 from degradation, MAP4K4 that regulates their activity via phosphorylation, and HSD17B11 that controls their action via a conformational change. Finally, using in vitro kinase assays on microfluidic microarrays, we pinpointed various downstream targets that are phosphorylated by the GCKIII kinases, with known functions in lipogenesis, lipolysis, and lipid secretion, as well as glucose uptake, glycolysis, hexosamine synthesis, and ubiquitination. Together, this study demonstrates that the members of the GCKIII kinase subfamily regulate hepatocyte lipid metabolism via common pathways. The results shed new light on the role of MST3, STK25, and MST4, as well as their interactions with PDCD10, MAP4K4, and HSD17B11, in the control of liver lipid homeostasis and metabolic dysfunction–associated steatotic liver disease susceptibility.

## Graphical Abstract

**Figure:**
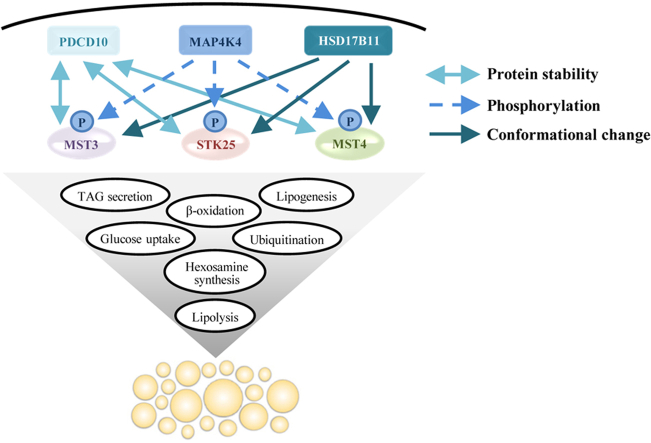

Metabolic dysfunction–associated steatotic liver disease (MASLD; previously referred to as nonalcoholic fatty liver disease) is defined by lipid accumulation in >5% of hepatocytes and is currently estimated to affect about 30% of the global population (1, 2). Although often clinically silent, MASLD can progress to metabolic dysfunction–associated steatohepatitis [MASH; formerly known as nonalcoholic steatohepatitis], which in addition to hepatic steatosis is characterized by liver inflammation and cell damage, with different degrees of fibrosis (3, 4). MASLD contributes to the pathogenesis of type 2 diabetes mellitus and cardiovascular disease (5). Patients with MASH also have an increased risk of developing hepatocellular carcinoma (HCC), which is the third major cause of cancer death and, in the Western world, the malignancy with the steepest increase in both incidence and mortality (6, 7). Importantly, to date, only resmetirom, a liver-targeted thyroid hormone receptor (THR)-beta selective agonist, has been approved by the FDA for the treatment of MASH; however, it is effective only in 30% of patients at 1-year follow-up, and no long-term data are yet available (8).

Ectopic deposition of fat within hepatocellular lipid droplets is considered a primary event in the initiation of MASLD, which triggers the activation of oxidative and endoplasmic reticulum (ER) stress, provoking local inflammation, fibrinogenesis, and apoptosis, which ultimately drive the disease progression to MASH (9, 10, 11). Liver lipid droplets participate in various cellular functions, such as lipid partitioning, cell signaling, protein quality control and storage, and interactions with other subcellular organelles (12). These outcomes are regulated by the composition of proteins that coat the surface of lipid droplets, which are altered in response to steatosis-inducing diets (13). Interestingly, the best-characterized genetic risk factors controlling the susceptibility to MASLD—*PNPLA3, HSD17B13, PLIN2* (also known as ADPR and adipophilin), and *MBOAT7*—encode proteins anchored to the hepatocellular lipid droplets (9, 12). Of note, liver-specific oligonucleotide-based therapies targeting *PNPLA3* and *HSD17B13* are currently being evaluated in clinical trials for the management of MASH (14).

Our recent translational studies have revealed that the STE20-type kinases comprising the GCKIII subfamily—MST3 (also known as STK24), STK25 (also known as YSK1 and SOK1), and MST4 (also known as STK26 and MASK)—associate with hepatocellular lipid droplets and regulate liver fat homeostasis and MASLD development (15). We reported a significant positive correlation between *MST3*, *STK25*, and *MST4* expression in human liver biopsies and all three individual lesions of the MASLD activity score (MAS; i.e., histological scores of hepatic steatosis, lobular inflammation, and ballooning degeneration) (16, 17, 18, 19). We also showed that the silencing or overexpression of the GCKIII kinases in human hepatocytes markedly reduces or aggravates, respectively, intracellular lipid accumulation and oxidative/ER stress (16, 17, 18, 19, 20). Moreover, *Stk25* knockout mice and mice treated with *Stk25*- or *Mst3*-targeting antisense oligonucleotides are protected against the deleterious effects of MASH-inducing diet, with livers displaying lowered steatosis, inflammatory infiltration, fibrosis, and cellular damage highlighted by ballooning and apoptosis (18, 21, 22, 23, 24).

Although these previous observations provide several lines of experimental support for a key role of MST3, STK25, and MST4 in the regulation of the hepatocellular lipotoxic milieu and MASLD susceptibility, their specific molecular mode of action, including binding partners, upstream elements, and downstream targets, remains elusive. This study aims to identify the signaling pathways through which the GCKIII kinases control ectopic lipid storage within human hepatocytes, potentially paving the way for innovative MASLD prevention and treatment strategies.

## Materials and methods

### Cell culture and transfection assays

Immortalized human hepatocytes [IHHs; a kind gift from B. Staels, the Pasteur Institute of Lille, University of Lille Nord de France, Lille, France; (25)] and LX-2 cells (human stellate cells; Millipore, Burlington, MA) were maintained as previously described (26, 27). THP-1 cells (human monocytic cells; American Type Culture Collection, Manassas, VA) were cultured as earlier reported (28) and differentiated into macrophages by the treatment with 100 nmol/l phorbol 12-myristate 13-acetate (Sigma-Aldrich, St. Louis, MO) for 48 h. The MycoAlert Mycoplasma Detection Kit (Lonza, Basel, Switzerland) was used to show that cells were free of mycoplasma contamination.

For RNA interference, cells were transfected with human *MST3*, *STK25*, *MST4*, *PDCD10*, *MAP4K4*, *GOLGA2*, *BAZ2A*, and/or *HSD17B11* siRNA (Thermo Fisher Scientific, Waltham, MA), or scrambled siRNA (Sigma-Aldrich), using Lipofectamine RNAiMax (Thermo Fisher Scientific). Notably, we observed significant interexperimental heterogeneity in knockdown efficiency (∼60–90% silencing of the individual GCKIII kinases across all experiments performed in IHHs; ∼60–80% in THP-1 cells, and ∼50–80% in LX-2 cells). For overexpression, cells were transfected with human *MST3, MST4, PDCD10, GOLGA2, BAZ2A, HSD17B11* (all *MYC*-tagged), and/or *STK25* (*FLAG*-tagged) expression plasmids, or corresponding empty control plasmids (GeneCopoeia, Nivelles, Belgium), using Lipofectamine 2000 (Thermo Fisher Scientific). The culture medium was replaced by fresh medium supplemented with 50 μmol/l oleic acid and 400 μmol/l palmitic acid (Sigma-Aldrich) 24 h after transfections for a subsequent 48 h incubation. To assess cell viability, the CellTiter-Blue Cell Viability Assay (Promega, Stockholm, Sweden) was used according to the manufacturer’s instructions.

### Assessment of lipid metabolism and oxidative/ER stress

Cells were stained with Bodipy 493/503 (Invitrogen, Carlsbad, CA) or Oil Red O (ORO; Sigma-Aldrich) to examine neutral lipid content, or dihydroethidium (DHE; Life Technologies, Grand Island, NY) for detection of superoxide radicals. To quantify ORO, 100% isopropanol was added to the cells, and the extracted dye was monitored spectrophotometrically at 500 nm. In parallel, cells were processed for immunofluorescence with anti-4-hydroxynonenal (4-HNE) or anti-8-oxoguanine (8-oxoG) antibodies to measure oxidative stress, or anti-C/EBP-homologous protein (CHOP) or anti-KDEL antibodies to investigate ER stress (see supplemental Table S1 for antibody information). Images were acquired using a Zeiss Axio Observer or LSM 700 microscope with the ZEN Blue software (Zeiss, Oberkochen, Germany). The labeled area was quantified in six randomly selected microscopic fields (×20) per well of the cell culture chamber using the ImageJ software (1.47v; National Institutes of Health, Bethesda, MD).

For analysis of fatty acid partitioning, cells were incubated in low glucose (5.5 mmol/l) Dulbecco's Modified Eagle Medium containing 1 mmol/l carnitine, 2% BSA, 0.5 mmol/l oleate, and 0.5 μCi/ml [1-^14^C]-oleate for 4 h. Cells were subjected to Folch extraction (29) and the [1-^14^C]-oleate incorporation into complex lipids was determined by resuspending the extracted lipids in chloroform/methanol (2:1), followed by the separation of lipids using thin-layer chromatography on silica gel plates. Radiolabeled triacylglycerol (TAG) was then detected by iodine vapor and quantified by a scintillation counter. TAGs secreted to the medium were examined after Folch extraction as described above. Fatty acid oxidation was assessed by adding an equal volume of 1 mol/l perchloric acid to the culture medium and measuring the liberated [1-^14^C]-CO_2_ trapped in an Eppendorf tube containing 1 mol/l sodium hydroxide.

The TAG content was measured in cell lysates using the Triglyceride Colorimetric Assay Kit (Cayman Chemical, Ann Arbor, MI) according to the manufacturer’s instructions. In addition, individual TAG and ceramide species were evaluated by lipidomics. In brief, lipids were extracted from cells using the BUME method (30); TAGs were analyzed using direct infusion on a QTRAP 5500 mass spectrometer (Sciex, Concord, Canada) equipped with a robotic nanoflow ion source (TriVersa NanoMate; Advion BioSciences, Ithaca, NJ) and ceramides were measured using ultrahighperformance LC-MS.

To measure retinol dehydrogenase activity, cells were incubated with 5 μmol/l all-trans-retinol (Thermo Fisher Scientific) for 8 h and harvested in PBS. Cells were then subjected to two freeze-thaw cycles and retinoic acid concentration was analyzed in cell lysate using the Human Retinoic Acid ELISA Kit (Cusabio, Houston, TX) according to the manufacturer’s instructions.

### Yeast two-hybrid analysis

Yeast two-hybrid (Y2H) screening was performed by Hybrigenics Services, S.A.S., Evry, France (http://www.hybrigenics-services.com). The coding sequences for *Homo sapiens* MST3 (NM_003576.5; amino acid positions 2 to 443) and MST4 (NM_016542.4; amino acid positions 2 to 416) were PCR-amplified and cloned into a pB27 plasmid as a C-terminal fusion to the LexA DNA-binding domain. The constructs were first checked by sequencing and then used as bait to screen a random-primed primary human hepatocytes cDNA library, which was constructed into a pP6 plasmid. The pB27 and pP6 plasmids derive from the original pBTM116 (31) and pGASGH (32) plasmids, respectively. The Y2H screen for STK25 has been previously described (26).

In total, 35.6 million clones (3-fold the complexity of the library) and 84.6 million clones (7-fold the complexity of the library) were screened for the LexA-MST3 and LexA-MST4 bait constructs, respectively, using a mating approach with YHGX13 (Y187 ade2-101::loxP-kanMX-loxP, matα) and L40ΔGal4 (mata) yeast strains as earlier reported (33). Using a medium lacking tryptophan, leucine, and histidine, 35 His+ colonies were selected for both constructs. The prey fragments of the positive clones were amplified by PCR and sequenced at their 5′ and 3′ junctions. The resulting sequences were used to identify the corresponding interacting proteins in the GenBank database (NCBI) using a fully automated procedure (34, 35). A confidence score (PBS, for predicted biological score) was attributed to each interaction as previously described (34). In brief, the PBS relies on two different levels of analysis. First, a local score accounts for the redundancy and independency of prey fragments, along with the distribution of reading frames and stop codons in overlapping fragments. Second, a global score considers the interactions found in all the screens conducted at Hybrigenics using the same library, representing the probability of an interaction being nonspecific. For practical use, the scores were divided into four categories, from A (highest confidence) to D (lowest confidence). A fifth and sixth category (E and F) specifically flags interactions involving highly connected prey domains previously found several times in screens performed on libraries derived from the same organism (E) and domains that have been confirmed as false positives of the technique (F). Finally, N/A refers to the interactions where the conventional PBS is not applicable as the local score cannot be set due to the structural features of the fragment. The PBS has been shown to positively correlate with the biological significance of the interactions (35, 36). However, even interactions with moderate confidence scores can be physiologically significant (37, 38).

### Analysis of liver biopsies from human participants

The *HSD17B11* mRNA expression was measured in liver biopsies from 62 Caucasian individuals (men, n = 35; women, n = 27), who were recruited from subjects undergoing laparoscopic abdominal surgery for Roux-en-Y bypass (n = 12), elective cholecystectomy (n = 41), or sleeve gastrectomy (n = 9). Total body fat was analyzed by dual X-ray absorptiometry, and liver fat content was assessed by single-proton magnetic resonance spectroscopy as earlier reported (39). Liver biopsies were collected during the surgery (between 08:00 and 10:00 am) after an overnight fast, instantly snap-frozen in liquid nitrogen, and stored at −80°C for further preparation. In liver biopsies, histological features were blindly evaluated by two specialized hepatopathologists in H&E- and ORO-stained sections using the well-validated MAS (i.e., histological scores of liver steatosis, inflammation, and ballooning) and liver fibrosis score, as recommended by the MASH Clinical Research Network classification system (40). Quantitative real-time PCR analysis on liver biopsies was performed as described below using the probes for *HSD17B11* (Hs00212226_m1) and 18S rRNA (Hs99999901_s1; Thermo Fisher Scientific), which span exon-exon boundaries to improve the specificity. For participant characteristics and details on inclusion/exclusion criteria, see supplemental Table S2 and Cansby *et al.* (16).

All investigations were approved by the Ethics Committee of the University of Leipzig, Germany (approval numbers 363-10-13122010 and 159-12-21052012) and conducted in accordance with the Declaration of Helsinki and Istanbul. Before taking part in this study, all patients gave written informed consent to use their data in anonymized form for research purposes.

### Phosphopeptide analysis and integration with human-genome-scale metabolic model

Custom peptide sequences were synthesized in situ on a μParaflo microfluidic microarray which consists of 3,968 3D chambers, where >400 protein kinase assays were carried out in triplicate (https://lcsciences.com/; LC Sciences, Houston, TX). Net phosphorylation signal for each potential substrate was determined by subtracting the signal for the negative control (i.e., serine or threonine substituted with alanine) from that for the corresponding kinase substrate sequence. Only sequences exhibiting a signal-to-noise ratio >3 and a coefficient of variation <0.5 were classified as detectable. A signal intensity of 300 was used to filter initial data, followed by the application of a false discovery rate (FDR) correction to account for multiple comparisons. Adjusted *P* values were calculated using the Benjamini-Hochberg method, with an FDR threshold of 0.01 applied to identify statistically significant phosphorylation events. The substrate data was then integrated with human-genome-scale metabolic model v1.10.0 using the GSAM package (41).

### Quantitative real-time PCR, immunoprecipitation, and Western blot

The RNeasy Lipid Tissue Mini Kit (Qiagen, Hilden, Germany) and the EZNA Total RNA Kit (Omega Bio-Tek, Norcross, GA) were used to isolate RNA from human liver biopsies and cultured human hepatocytes, respectively. The following cDNA synthesis was performed using the High-Capacity cDNA Reverse Transcription Kit (Thermo Fisher Scientific). Relative quantification was conducted using the QuantStudio 6 Flex Real-Time PCR System (Thermo Fisher Scientific) or the CFX Connect Real-Time System (Bio-Rad, Hercules, CA). Relative quantities of target transcripts were calculated from duplicate samples after normalization of the data to the endogenous control, 18S rRNA (Thermo Fisher Scientific). Immunoprecipitation was carried out with Dynabeads Protein G magnetic beads (Thermo Fisher Scientific) according to the manufacturer’s instructions. Western blot analysis was performed as previously reported (42) using 4–12% tris-glycine and 4–12% bis-tris gels (Thermo Fisher Scientific) for native and sodium dodecyl sulfate gel electrophoresis, respectively (see supplemental Table S1 for antibody information).

### Statistical analysis

One-way ANOVA with a two-sample Student’s *t* test for post hoc analysis was used to evaluate statistical significance between the groups. Differences were considered statistically significant at *P* < 0.05. Correlation between *HSD17B11* expression in human liver biopsies and hepatic lipid content as well as MAS and fibrosis score was examined by Spearman’s rank correlation analysis. All statistical analyses were performed using SPSS statistics (27v; IBM Corporation, Armonk, NY).

## Results

### MST3, STK25, and MST4 play overlapping but nonredundant roles in the control of hepatocellular lipotoxicity

**Fig. 1:**
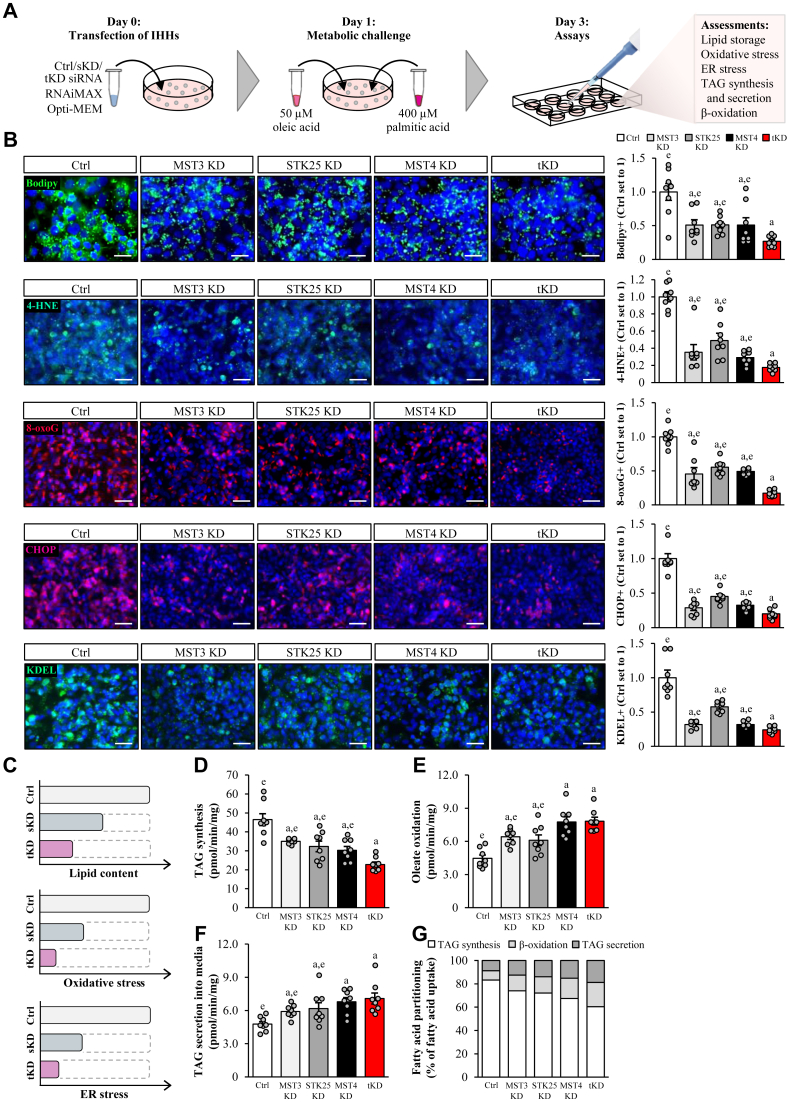
Single knockdown of MST3, STK25, or MST4 suppresses fatty acid–induced lipotoxicity in human hepatocytes to a similar degree, with the decrease in ectopic lipid accumulation and metabolic stress being slightly more pronounced in triple-deficient hepatocytes. IHHs were transfected with *MST3*, *STK25, MST4*, and/or NTC siRNA, and incubated with oleic and palmitic acid for 48 h posttransfection. A: Schematic illustration of the study design. B: Representative images of cells stained with Bodipy (green) or processed for immunofluorescence with anti-4-HNE (green), anti-8-oxoG (red), anti-CHOP (red), or anti-KDEL (green) antibodies; nuclei stained with DAPI (blue). Quantification of the staining. Scale bar: 50 μm. C: Schematic illustration of the impact of single versus triple knockdown of the GCKIII kinases on hepatocellular lipotoxicity. D: TAG synthesis from [^14^C]-labeled oleic acid. E: Oxidation of [^14^C]-labeled oleic acid. F: Secretion of [^14^C]-labeled TAG into the medium. G: Relative fatty acid partitioning based on the results shown in (D–F). Data are mean ± SEM from 8 cell culture wells per group. For (B and D–G), representative results from 2 to 3 independent experiments are shown.^a^*P* < 0.05 versus control; ^e^*P* < 0.05 versus tKD. 4-HNE, 4-hydroxynonenal; 8-oxoG, 8-oxoguanine; CHOP, C/EBP-homologous protein; Ctrl, control; KD, knockdown; DAPI, 4',6-diamidino-2-phenylindole; NTC, nontargeting control; IHH, immortalized human hepatocyte; sKD, single knockdown; TAG, triacylglycerol; tKD, triple knockdown; Y2H, yeast two-hybrid.

We set out to determine whether there was a possible additive or synergistic impact of the combinatorial silencing of all three GCKIII kinases in regulating hepatocellular lipotoxicity by comparing single versus triple siRNA knockdown of MST3, STK25, and/or MST4 in IHHs. In all experiments, transfected cells were treated with a mixture of oleic and palmitic acid, which efficiently induces steatosis in vitro and replicates the metabolic milieu in high-risk individuals (Fig. 1A for the schematic illustration of the study design). All three siRNAs effectively reduced the target gene expression when used separately or in combination (supplemental Fig. S1). Notably, the silencing of each kinase had no impact on the protein abundance of the other two kinases (supplemental Fig. S1).

In line with our previous investigations (16, 17, 19), we observed that hepatocytes transfected with *MST3*, *STK25*, or *MST4* siRNA displayed significantly decreased intracellular fat storage assessed by staining with the lipophilic dyes Bodipy 493/503 and ORO as well as colorimetric analysis of TAG content, and reduced oxidative and ER stress quantified by immunostaining for 4-hydroxynonenal (measures lipid peroxidation), 8-oxoguanine (detects oxidative DNA damage), C/EBP-homologous protein (a marker for ER stress–induced cell death), and KDEL (a signal motif for ER retrieval), compared to cells transfected with nontargeting control (NTC) siRNA (Fig. 1B, C and supplemental Fig. S2A, B). We also found that the depletion of all three kinases was more effective in preventing ectopic fat accumulation and oxidative/ER stress compared with hepatocytes treated with single siRNA (Fig. 1B, C and supplemental Fig. S2A, B). Mechanistically, both the single and triple silencing of MST3, STK25, and/or MST4 inhibited TAG synthesis and augmented the rates of mitochondrial β-oxidation and VLDL-TAG secretion (Fig. 1D–G). Consistent with the impact on fat storage and metabolic stress, these alterations in lipid partitioning were most pronounced in triple-deficient hepatocytes; however, the differences compared with the single knockdown were relatively minor (Fig. 1D–G). Notably, the silencing of MST3, STK25, and/or MST4 had no effect on the viability of IHHs (supplemental Fig. S3A).

Recent evidence shows that, similar to hepatocytes, liver nonparenchymal cells are susceptible to lipotoxic damage and causally contribute to MASH initiation and progression by stimulating inflammation and fibrogenesis (43, 44). Interestingly, *MST3*, *STK25*, and/or *MST4* depletion did not alter fatty acid–induced lipid deposition or oxidative stress in THP-1–derived human macrophages or LX-2 human hepatic stellate cells (supplemental Figs. S3B, C and S4).

**Fig. 2:**
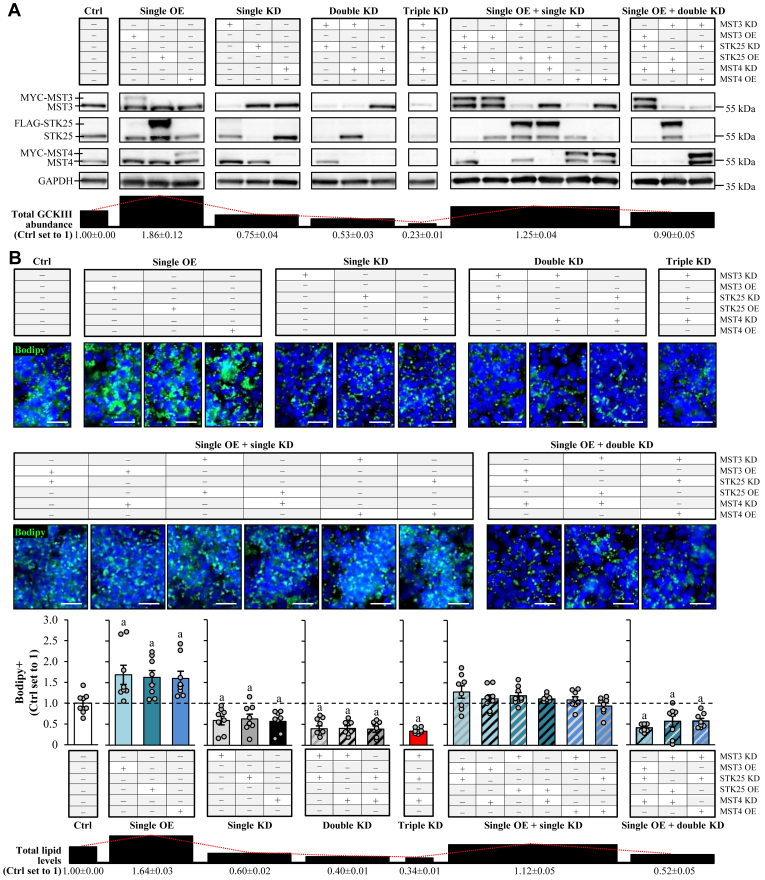
Total abundance of the GCKIII kinases is positively correlated with the hepatocellular lipid content. IHHs were transfected with different combinations of *MST3*, *STK25*, *MST4* siRNA, and/or with *MYC-MST3*, *FLAG-STK25*, *MYC-MST4* expression plasmids as indicated. Control cells were transfected with NTC siRNA and/or empty control plasmids. All cells were incubated with oleic and palmitic acid for 48 h posttransfection. A: Cell lysates were analyzed by Western blot using antibodies specific for MST3, STK25, or MST4. Protein levels were quantified by densitometry; representative Western blots are shown with GAPDH used as a loading control (quantifications are presented in supplemental Fig. S5). The average total GCKIII abundance for each set of the transfection combination is demonstrated at the bottom. B: Representative images of cells stained with Bodipy (green); nuclei stained with DAPI (blue). Quantification of the staining. The average lipid levels for each set of the transfection combination are demonstrated at the bottom. Scale bar: 50 μm. Data are mean ± SEM from 3 (A) or 8 (B) cell culture wells per group. ^a^*P* < 0.05 versus control. IHH, immortalized human hepatocyte; Ctrl, control; KD, knockdown; OE, overexpression; NTC, nontargeting control; tKD, triple knockdown; NTC, nontargeting control; DAPI, 4',6-diamidino-2-phenylindole.

Next, we studied the impact of varying the total GCKIII protein dosage on hepatocellular lipotoxicity by overexpressing and/or silencing MST3, STK25, and/or MST4 in different combinations in oleate- and palmitate-treated IHHs. In line with our previous results (Fig. 1B, C and supplemental Fig. S2) (16, 17, 19), we observed increased or decreased hepatocellular fat storage when individual kinases were overexpressed or knocked down, respectively, compared to the corresponding controls (Fig. 2 and supplemental Fig. S5). The lipid content–lowering effect of the double silencing of MST3, STK25, and/or MST4 was slightly augmented compared with the single knockdown of each kinase and was similar to that observed in triple-deficient hepatocytes (Fig. 2 and supplemental Fig. S5). Importantly, when the transfection with the *MST3*, *STK25*, or *MST4* expression plasmid, which enhanced target protein abundance about 3- to 4-fold, was combined with the single silencing of any of the other two kinases (e.g., knockdown of MST3, overexpression of MST4), the intracellular fat levels were elevated to the quantities detected in the control cells (Fig. 2 and supplemental Fig. S5). However, overexpressing the third kinase in hepatocytes with the combined silencing of the two other kinases (e.g., knockdown of MST3 and STK25, overexpression of MST4) was insufficient to restore the lipid content to normal levels (Fig. 2 and supplemental Fig. S5).

Together, the lack of additive or synergistic effects of the combined depletion of the three GCKIII kinases, compared with individual protein knockdown, suggests that MST3, STK25, and MST4 operate in the same signaling pathway and/or share mechanism(s) of action in regulating lipid storage in hepatocytes. Furthermore, our results reveal that hepatocellular fat content correlates with total GCKIII protein abundance, thereby implying that the GCKIII kinases, to some extent, can functionally compensate for the lack of each other.

### PDCD10 controls hepatocellular lipid accumulation by protecting the GCKIII kinases from degradation

**Fig. 3:**
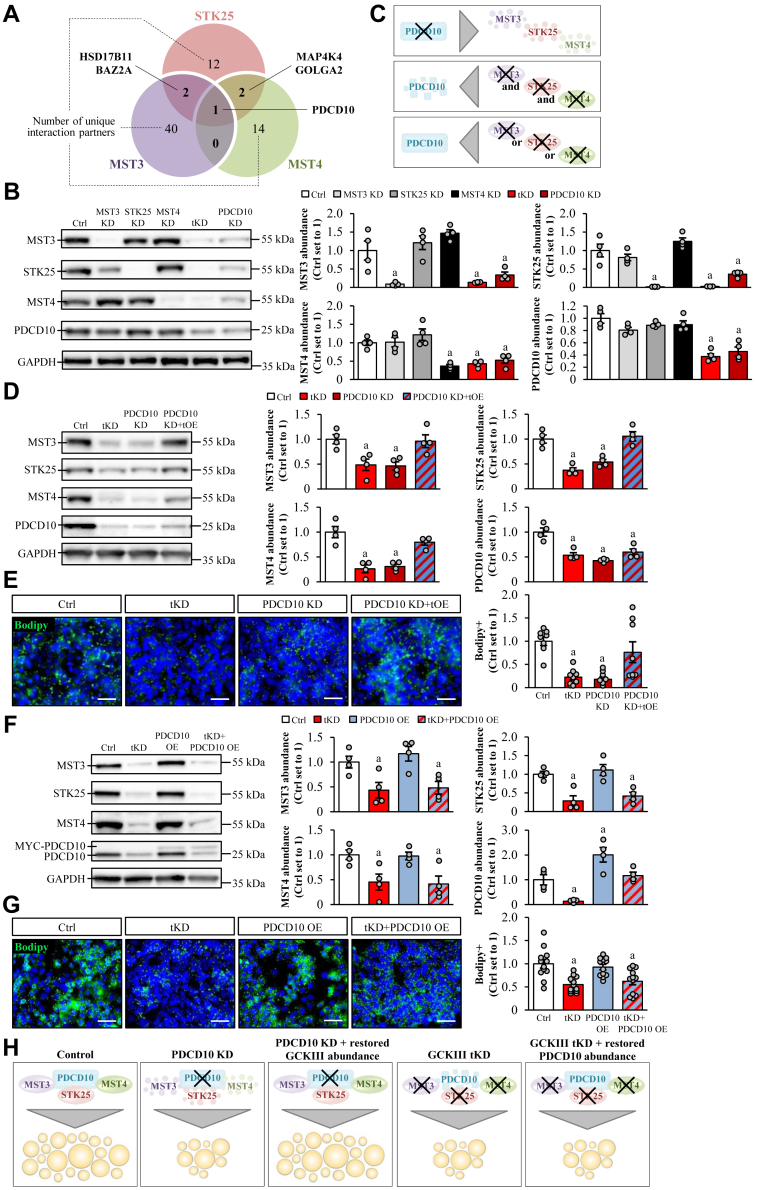
MST3, STK25, and MST4 interact with PDCD10, which regulates the hepatocellular lipid storage by controlling the stability of the GCKIII kinases. A: Venn diagram showing the number of shared and unique interaction partners of the GCKIII kinases detected by ULTImate Y2H screen of a primary human hepatocyte cDNA library. For (B-H), IHHs were transfected with different combinations of *MST3*, *STK25*, *MST4*, *PDCD10* siRNA, and/or with *MYC-MST3*, *FLAG-STK25*, *MYC-MST4*, and *MYC-PDCD10* expression plasmids as indicated. Control cells were transfected with NTC siRNA and/or empty control plasmids. All cells were incubated with oleic and palmitic acid for 48 h posttransfection. (B, D, and F) Cell lysates were analyzed by Western blot using antibodies specific for MST3, STK25, MST4, or PDCD10. Protein levels were quantified by densitometry; representative Western blots are shown with GAPDH used as a loading control. C: Schematic illustration of the impact of PDCD10 or GCKIII knockdown on protein stability. (E and G) Representative images of cells stained with Bodipy (green); nuclei stained with DAPI (blue). Quantification of the staining. Scale bar: 50 μm. (H) Schematic illustration of the impact of modifying PDCD10 and/or GCKIII abundance on hepatocellular lipid accumulation. Data are mean ± SEM from 4 (B, D, and F) or 8–12 (E and G) cell culture wells per group. For (B and D–G), representative results from 2 to 3 independent experiments are shown. ^a^*P* < 0.05 versus control. DAPI, 4',6-diamidino-2-phenylindole; Ctrl, control; IHH, immortalized human hepatocyte; KD, knockdown; OE, overexpression; NTC, nontargeting control; tKD, triple knockdown; tOE, triple overexpression; Y2H, yeast two-hybrid.

We next sought to identify the interaction partners of MST3, STK25, and MST4 by using a genome-wide Y2H screen of the human hepatocyte library. Based on the evidence of their shared mode of action, we primarily focused on analysis of proteins that were found to bind to more than one GCKIII kinase (Fig. 3A and supplemental Tables S3–S5). Notably, we did not detect any direct interaction between MST3, STK25, and MST4 by Y2H screens.

Somewhat surprisingly, we discovered that only PDCD10 [also known as CCM3; a scaffold protein known to tether different targets to the multisubunit signaling complex STRIPAK (45)], bound to all three GCKIII kinases (Fig. 3A). To evaluate the impact of the interaction with PDCD10 on the lipotoxic effect of MST3, STK25, and MST4, we silenced PDCD10 in oleate- and palmitate-treated IHHs by siRNA approach. We found that the protein levels of MST3, STK25, and MST4 were markedly reduced upon PDCD10 knockdown (Fig. 3B), which aligns with previous observations in extrahepatic tissues (46, 47, 48, 49, 50). Conversely, there was a striking decrease in PDCD10 protein amount in hepatocytes deficient in all three GCKIII kinases, while the stability of PDCD10 was not affected in cells where only one kinase was depleted (Fig. 3B).

Next, we assessed the effect of altered PDCD10 expression on hepatocellular lipid content. In hepatocytes transfected with *PDCD10* siRNA, lipid storage was diminished to the levels seen in cells with the combined knockdown of MST3, STK25, and MST4 (Fig. 3D, E). This was expected due to the lower stability of the GCKIII kinases in PDCD10-deficient cells (Fig. 3B) and lower lipid levels in cells where the GCKIII kinases were silenced (Fig. 2B). Importantly, rescuing MST3, STK25, and MST4 protein levels in PDCD10-depleted hepatocytes via overexpression restored intracellular lipid content to that observed in the control cells (Fig. 3D, E). In contrast, restoring PDCD10 protein levels in hepatocytes depleted of all three GCKIII kinases failed to impact on lipid storage (Fig. 3F, G).

Together, these results indicate that the stability of the GCKIII kinases and PDCD10 is mutually dependent and that PDCD10 controls hepatocellular lipid accumulation solely by protecting MST3, STK25, and MST4 proteins from degradation (Fig. 3C, H).

### MAP4K4 regulates hepatocellular lipotoxicity via phosphorylation of the GCKIII kinases

In addition to PDCD10, several other proteins were observed to interact with multiple GCKIII kinases. These included MAP4K4 (also known as HGK and NIK) which is an STE20-type kinase that controls a wide range of cellular functions, such as migration, proliferation, and stress responses (51), the Golgi matrix protein GOLGA2 (also known as GM130 and Golgin A2) which facilitates vesicle fusion (52), BAZ2A which is involved in transcriptional regulation through recruitment of histone-modifying enzymes and DNA methyltransferases (53), and HSD17B11 (also known as 17-beta-HSDXI, RETSDR2, and PAN1B) which is a hydroxysteroid dehydrogenase regulating steroid hormone metabolism (54) (Fig. 3A). Next, we examined the effect of loss or gain of function of these interaction partners on the GCKIII kinase protein abundance and lipid homeostasis in human hepatocytes.

**Fig. 4:**
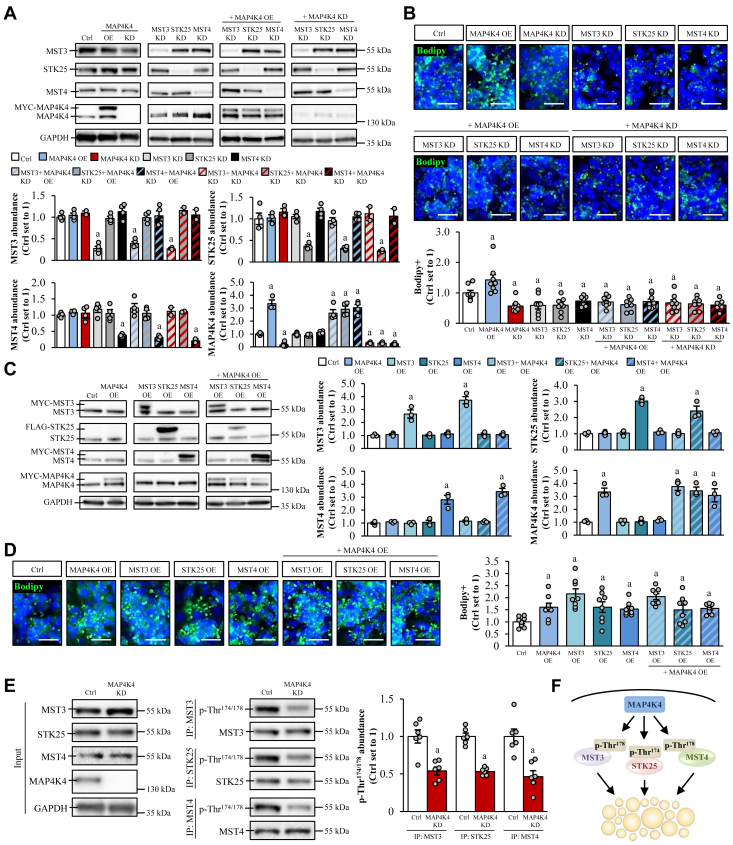
MAP4K4 controls hepatocellular lipid accumulation via activation of the GCKIII kinases. IHHs were transfected with different combinations of *MST3*, *STK25*, *MST4*, *MAP4K4* siRNA, and/or with *MYC-MST3*, *FLAG-STK25*, *MYC-MST4*, and *MYC-MAP4K4* expression plasmids as indicated. Control cells were transfected with NTC siRNA and/or empty control plasmids. All cells were incubated with oleic and palmitic acid for 48 h posttransfection. (A and C) Cell lysates were analyzed by Western blot using antibodies specific for MST3, STK25, MST4, or MAP4K4. Protein levels were quantified by densitometry; representative Western blots are shown with GAPDH used as a loading control. (B and D) Representative images of cells stained with Bodipy (green); nuclei stained with DAPI (blue). Quantification of the staining. Scale bar: 30 μm. E: Cell lysates were immunoprecipitated with Dynabeads Protein G magnetic beads and subjected to Western blot using antibodies specific for MST3, STK25, MST4, MAP4K4, or phospho-MST3/-STK25/-MST4 (Thr^174/178^). Protein levels were quantified by densitometry; representative Western blots are shown with GAPDH used as a loading control. F: Schematic illustration of the role of MAP4K4 in GCKIII-mediated lipid accumulation within hepatocytes. For (E), representative results from 2 independent experiments are shown. Data are mean ± SEM from 3-6 (A, C, and E) or 8 (B and D) cell culture wells per group. ^a^*P* < 0.05 versus control. Ctrl, control; DAPI, 4',6-diamidino-2-phenylindole; IP, immunoprecipitation; IHH, immortalized human hepatocyte; KD, knockdown; OE, overexpression; NTC, nontargeting control.

We found that the knockdown of MAP4K4 significantly reduced lipid content in IHHs treated with oleic and palmitic acid and, reciprocally, MAP4K4 overexpression rendered hepatocytes more susceptible to fat deposition (Fig. 4A–D), which is in line with our previous studies (55). We also showed that the combined depletion or overexpression of MAP4K4 and the GCKIII kinases resulted in a similar alteration of hepatocellular lipid accumulation as compared with the changed abundance of each of these proteins individually (Fig. 4A–D), indicating that MAP4K4, MST3, STK25, and MST4 operate in the same signaling pathway. Importantly, the effect of the MAP4K4 overexpression was blocked in hepatocytes where any of the three GCKIII kinases were knocked down (Fig. 4A, B). In addition, the amount of phospho-MST3 (Thr^178^), phospho-STK25 (Thr^174^), and phospho-MST4 (Thr^178^; active forms) was about 2-fold lower in MAP4K4-deficient hepatocytes, with no differences in the levels of the corresponding total proteins (Fig. 4E). Together, these results suggest that MAP4K4 acts upstream of the GCKIII kinases, regulating their activity via phosphorylation (Fig. 4F).

We observed no alterations in fatty acid–induced lipid accumulation in IHHs when GOLGA2 or BAZ2A were overexpressed or knocked down (supplemental Figs. S6A, B and S7A, B). Furthermore, fat storage was increased or decreased to a similar extent by overexpressing or silencing of the GCKIII kinases, respectively, in GOLGA2- or BAZ2A-deficient hepatocytes compared with the corresponding control cells (supplemental Figs. S6C–F, and S7C–F). Thus, we found no evidence that the interaction with GOLGA2 or BAZ2A proteins is critical in the regulation of hepatocellular lipotoxicity mediated by MST3, STK25, and MST4.

### Interaction with HSD17B11 is instrumental for mediating the lipotoxicity-modifying function of the GCKIII kinases

**Fig. 5:**
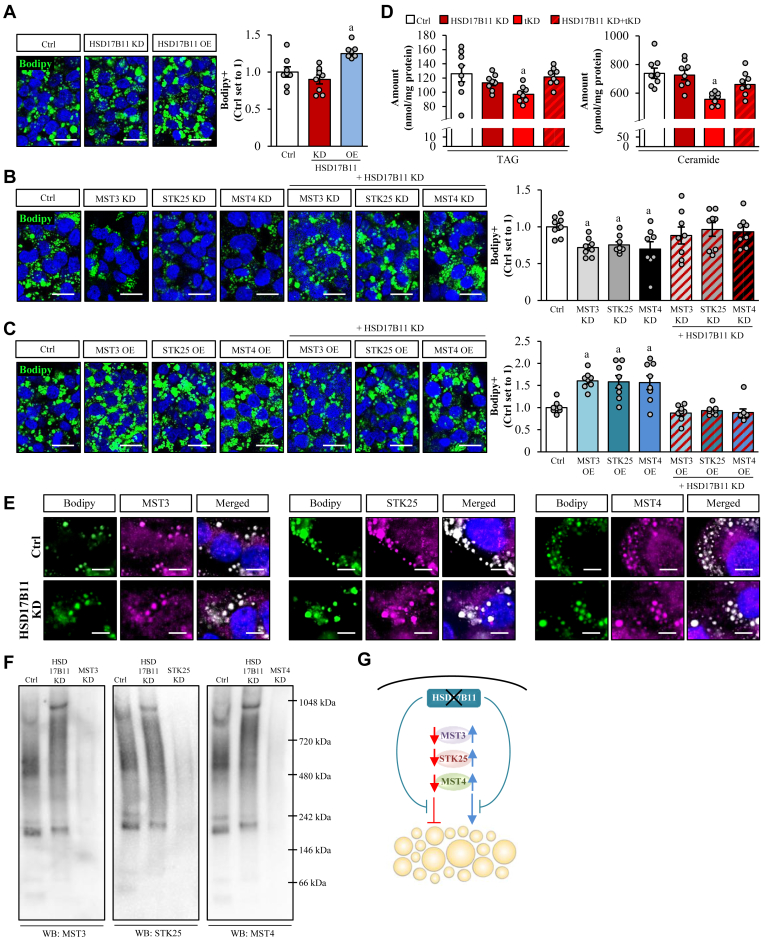
HSD17B11 mediates the lipotoxicity-modifying activity of the GCKIII kinases via a conformational change. IHHs were transfected with different combinations of *MST3*, *STK25*, *MST4*, *HSD17B11* siRNA, and/or with *MYC-MST3*, *FLAG-STK25*, *MYC-MST4*, *MYC-HSD17B11* expression plasmids as indicated. Control cells were transfected with NTC siRNA and/or empty control plasmids. All cells were incubated with oleic and palmitic acid for 48 h posttransfection. A-C: Representative images of cells stained with Bodipy (green); nuclei stained with DAPI (blue). Quantification of the staining. Scale bar: 25 μm. D: Lipidomic analysis by ultrahigh performance LC-MS. The abundance of the individual molecular lipid species is presented in supplemental Table S6. E: Representative images of cells stained with Bodipy (green) and processed for immunofluorescence with anti-MST3, anti-STK25, or anti-MST4 antibodies (violet); merged image shows colocalization in white with nuclei stained with DAPI (blue). Scale bar: 10 μm. F: Cell lysates were analyzed by native gel electrophoresis followed by immunoblotting using antibodies specific for MST3, STK25, or MST4. G: Schematic illustration of the role of HSD17B11 in GCKIII-mediated lipotoxicity. Data are mean ± SEM from 8 cell culture wells per group. For (A-B, and F), representative results from 2 independent experiments are shown. ^a^*P* < 0.05 versus control. Ctrl, control; DAPI, 4',6-diamidino-2-phenylindole; IHH, Immortalized human hepatocyte; KD, knockdown; OE, overexpression; NTC, nontargeting control; tKD, triple knockdown; WB, Western blot.

Next, we explored the potential role of the interaction partner HSD17B11 in the GCKIII-induced lipotoxicity. We detected no significant differences in lipid accumulation or superoxide radical (O^•−^) content quantified by Bodipy 493/503 or DHE staining, respectively, in oleate- and palmitate-treated IHHs transfected with *HSD17B11* siRNA versus NTC siRNA, while the overexpression of HSD17B11 increased both fat storage and oxidative stress (Fig. 5A, supplemental Fig. S8A, and S9A). Remarkably, we found that the impact of MST3, STK25, or MST4 silencing or overexpression on hepatocellular lipid levels measured by Bodipy 493/503 and oxidative damage assessed by DHE was completely blocked in HSD17B11-deficient cells (Fig. 5B, C, supplemental Figs. S8B, C, and S9B, C). Lipidomic analysis by ultrahigh performance LC-MS further confirmed hindered reduction in TAGs, which are the major constituents of the neutral core of lipid droplets, in hepatocytes where the knockdown of the GCKIII kinases was combined with the depletion of HSD17B11 (Fig. 5D and supplemental Table S6). We also observed a similar pattern for total ceramides, which have been specifically implicated in the development of MASLD (Fig. 5D and supplemental Table S6) (56). Of note, the overexpression of HSD17B11 had no significant influence on the lipid-lowering or lipid-elevating effect seen in hepatocytes with deceased or enhanced abundance of the individual GCKIII kinases, respectively (supplemental Fig. S10).

We detected a significant positive correlation between *HSD17B11* mRNA expression in human liver biopsies and the hepatic fat content analyzed by magnetic resonance spectroscopy as well as the severity of MASH assessed by MAS (i.e., histological scores of liver steatosis, inflammation, and ballooning) and hepatic fibrosis stage as recommended by the MASH Clinical Research Network (40) (supplemental Fig. S11 and, supplemental Table S2).

Mechanistically, we found that the silencing of HSD17B11 did not impact the stability or activity of the GCKIII kinases, as evidenced by equal abundance of total and phospho-MST3 (Thr^178^), phospho-STK25 (Thr^174^), and phospho-MST4 (Thr^178^; active forms) in IHHs transfected with *HSD17B11* siRNA versus NTC siRNA (supplemental Figs. S8A and S12). Furthermore, the confinement of MST3, STK25, and MST4 to the surface of hepatocellular lipid droplets was unaffected by HSD17B11 knockdown (Fig. 5E). Interestingly, native gel electrophoresis showed an altered banding pattern with higher apparent molecular weight forms of all three GCKIII kinases in HSD17B11-deficient hepatocytes than the control cells, suggesting conformational change and assembly of MST3, STK25, and MST4 into a large complex or protein aggregates in the absence of HSD17B11 (Fig. 5F). Of note, the silencing of MST3, STK25, or MST4 had no effect on the enzymatic activity of HSD17B11 measured by conversion of retinol to retinoic acid (supplemental Fig. S13).

Together, our results indicate that HSD17B11 controls the lipotoxicity-modifying activity of the GCKIII kinases via a conformational change (Fig. 5G).

### Substrate profiling of the GCKIII kinases reveals targets linked to MASLD susceptibility

**Fig. 6:**
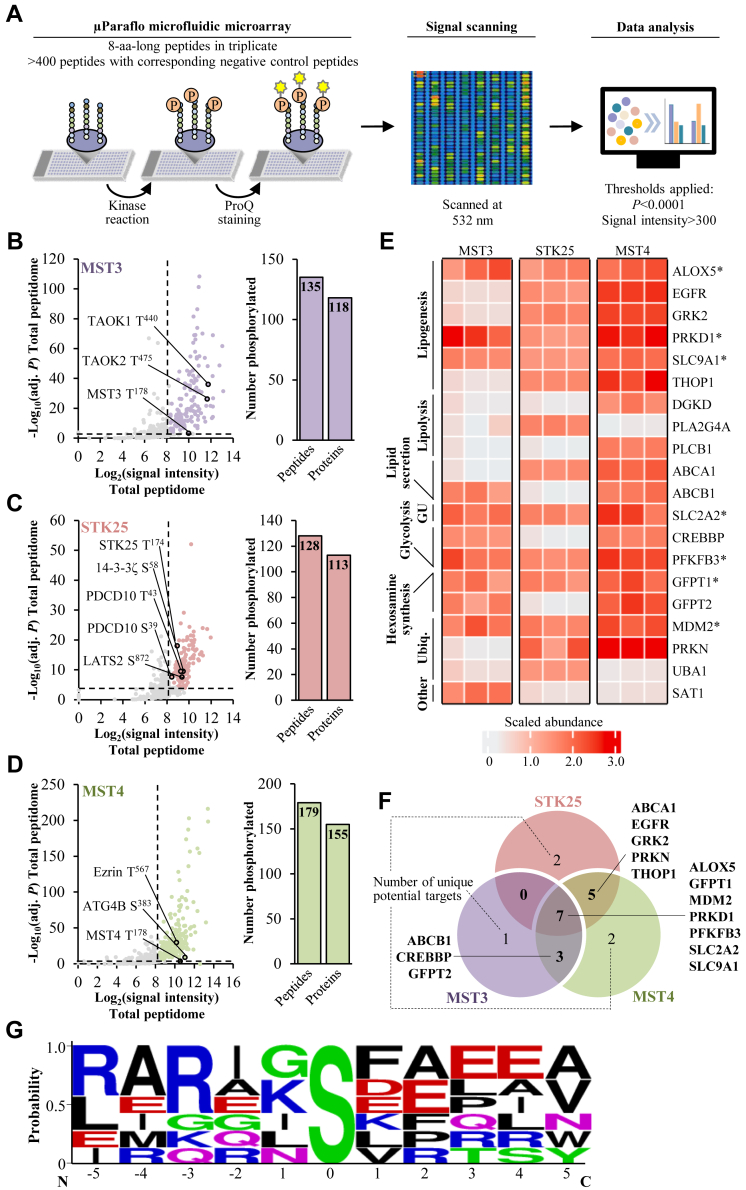
Substrate profiling of the GCKIII kinases identifies targets linked to MASLD susceptibility. A: Schematic illustration of the experimental design. B-D: Scatter plots of the log-transformed signal intensity and significance of differentially enriched phosphopeptides. Initial data were filtered using a signal intensity threshold of 300 (vertical dashed lines), and statistically significant phosphorylation events were identified using FDR correction with a 0.01 threshold (horizontal dashed lines). Previously known substrates for each kinase are indicated in the graphs. Bar graphs show the total number of differentially phosphorylated unique peptides and proteins. E: Heat map of the scaled abundance of significantly changed phosphoproteins integrated with genome-scale metabolic model Human1 (63). The known functions of the substrates are denoted on the left. Stars indicate substrates that were detected in the screens for all three kinases. F: Venn diagram showing the number of shared and unique targets of MST3, STK25, and MST4 in the subset of significantly changed phosphoproteins integrated with genome-scale metabolic model Human1. G: Consensus sequences of the subset of significantly changed phosphoproteins integrated with genome-scale metabolic model Human1 detected in the screens for all three kinases, using the WebLogo application (64). The residue position in relation to the phosphorylation site is shown on the *x*-axis and the information content is shown on the *y*-axis. Polar amino acids (G, S, T, Y, and C) are shown in green, neutral amino acids (Q and N) are shown in purple, basic amino acids (K, R, and H) are shown in blue, acidic amino acids (D and E) are shown in red, and hydrophobic amino acids (A, V, L, I, P, W, F, and M) are shown in black. aa, amino acids; FDR, false discovery rate; GU, glucose uptake; MASLD, metabolic dysfunction–associated steatotic liver disease; Ubiq., ubiquitination.

To characterize the phosphorylation targets of the GCKIII proteins, we performed in vitro kinase assays on μParaflo microfluidic microarrays with over 400 human peptides (Fig. 6A for the schematic illustration of the study design). In total, between 128 and 179 distinct peptides were identified as substrates for each kinase (signal intensity>300, FDR<0.01), corresponding to 113 and 155 unique proteins, respectively, including previously known targets (Fig. 6B–D) (57, 58, 59, 60, 61, 62). Notably, none of the proteins detected by the substrate profiling were recognized as interaction partners of the GCKIII kinases by Y2H screening, with the exception of PDCD10 (phosphorylated by STK25 at Ser^39^ and Thr^43^ residues; Fig. 6C). This was not unexpected as most phosphorylation events are intrinsically transient and, thus, the two methods are considered to be complementary rather than interchangeable.

To understand the GCKIII-dependent regulation of hepatocellular metabolism, we next integrated the results obtained from the substrate screen with the genome-scale metabolic model Human1 (63), including only proteins known to be expressed in the liver (65). This analysis revealed 20 targets in total, which were phosphorylated by one or several of the GCKIII kinases, with described functions in lipogenesis, lipolysis, and lipid secretion, as well as glucose uptake, glycolysis, hexosamine synthesis, and ubiquitination (Fig. 6E and supplemental Table S7). In this dataset, seven proteins were identified as phosphorylation substrates for all three GCKIII kinases, representing the candidates most likely to constitute the common pathway of MST3, STK25, and MST4 (Fig. 6F). Interestingly, all these shared targets (ALOX5, GFPT1, MDM2, PRKD1, PRKFB3, SLC2A2, and SLC9A1) have previously been linked to the regulation of liver lipotoxicity and MASLD susceptibility in human and/or mouse models (supplemental Table S7) (66, 67, 68, 69, 70, 71, 72). The seven phosphosites have been annotated in PhosphoSitePlus (73); however, the functional implication of these phosphorylation events has not been described. Alignment of the phosphosites of the shared targets using the WebLogo software (64) identified a strong preference for serine as the phosphoacceptor residue with a high variability in the amino acid sequences surrounding the site of phosphorylation (Fig. 6*G*).

## Discussion

Ectopic lipid accumulation in liver is a hallmark feature of MASLD and precursor for the progression to MASH. Many proteins have been reported to be involved in hepatic lipid homeostasis, yet the mechanisms that underpin the development of steatosis remain to be resolved. In this study, we explored the mode of action of the STE20 kinases comprising the GCKIII subfamily—MST3, STK25, and MST4—in the regulation of hepatocellular lipotoxicity. The impact of single versus combined silencing of the GCKIII kinases on lipid accumulation and oxidative/ER stress was assessed in human hepatocytes, and their interaction partners, upstream elements, and downstream targets were characterized.

Our systematic examination of the GCKIII subfamily revealed that the single knockdown of MST3, STK25, or MST4 resulted in a comparable reduction in hepatocellular lipid content and metabolic stress, implying overlapping but nonredundant roles for these kinases in the control of lipotoxicity. Interestingly, we observed no additive or synergistic effects when all three GCKIII kinases were simultaneously silenced, indicating that MST3, STK25, and MST4 likely operate in the same signaling pathway and/or employ a shared mechanism of action that is not substantially enhanced by combined depletion. While the mammalian genome encodes three GCKIII kinases, which possess a highly conserved catalytic domain [95% similarity in the amino acid sequence among the human proteins; (15)], lower organisms such as *Caenorhabditis elegans* have only one homolog of MST3, STK25, and MST4 [GCK-1; (74)]. The evolutionary expansion of a gene family typically reflects essential functional diversification required in higher eukaryotes; however, the GCKIII kinases appear to phenocopy the effect of each other on hepatocellular lipotoxicity. Of note, MST3, STK25, and MST4 are ubiquitously expressed and implicated in a diverse range of complex cellular processes including the regulation of Golgi integrity, cytoskeletal organization, polarity, proliferation, migration, and apoptosis across various cell types such as HeLa (cervical cancer cells), human embryonic kidney 239 (HEK293) cells, COS-7 (kidney fibroblasts), MCF-7 (mammary epithelium), tissue macrophages, and neurons (15, 75, 76). Thus, it is plausible that the role of MST3, STK25, and MST4 kinases differs in extrahepatic tissues or in pathways that we did not examine.

Protein-protein interaction is a major mechanism that regulates enzyme activity. By using a genome-wide Y2H screen of the human hepatocyte library, we identified the scaffold protein PDCD10 as an interaction partner of all three GCKIII kinases. We also found that in hepatocytes, the stability of the GCKIII kinases and PDCD10 was interdependent, where the silencing of PDCD10 significantly reduced the protein levels of MST3, STK25, and MST4, while the combined depletion of the GCKIII kinases, but not the knockdown of each of them individually, lowered the abundance of PDCD10. These results are consistent with earlier reports showing that PDCD10 silencing destabilizes the GCKIII kinases in SaOS2 cells (human osteoblasts) leading to Golgi disassembly, HeLa cells attenuating apoptosis, human umbilical vein endothelial cells diminishing adhesion capacity, and PC-3 cells (human prostate cancer cells) hindering proliferation (46, 47, 48, 49, 50). To this end, here we demonstrated for the first time that PDCD10 knockdown antagonized ectopic lipid accumulation in human hepatocytes by decreasing the protein amounts of the GCKIII kinases. Mechanistically, PDCD10 has been shown to block ubiquitination and subsequent proteasome-dependent degradation of MST3, STK25, and MST4 in SaOS2 and HeLa cells (46, 48); however, the pathways used by PDCD10 to control the protein levels of these kinases in hepatocytes still remain elusive. Interestingly, in addition to being detected in Y2H screens as an interaction partner of the GCKIII kinases, PDCD10 was also identified as the phosphorylation target for STK25 by in vitro kinase assays. The sites phosphorylated by STK25 (Ser^39^ and Thr^43^) have been annotated in PhosphoSitePlus (73); however, the functional significance of these modifications remains unknown and further studies are warranted to investigate whether the phosphorylation of PDCD10 protein is important for stabilizing PDCD10 itself and/or the GCKIII kinases.

MAP4K4, an STE20-type kinase belonging to the GCKIV subfamily, also interacts with STK25 and MST4, as determined by the Y2H screens. Our data demonstrated that MAP4K4 facilitated hepatocellular lipid accumulation by phosphorylating all three GCKIII kinases. These results are aligned with our earlier study showing that the silencing of MAP4K4, similar to the knockdown of MST3, STK25, and/or MST4, protects human hepatocytes against lipotoxic damage (55). Furthermore, a higher protein abundance of MAP4K4 has been found in the livers of high-fat versus chow diet–fed mice, which is paralleled by an increase in the phosphorylation of MST3 and STK25 (Thr^178^ and Thr^174^, respectively) (21, 55, 77). MAP4K4 has previously been described as an upstream activator of the c-Jun N-terminal kinase and extracellular signal–regulated kinase cascade in some but not all cell types (78). Notably, endogenous MAP4K4 protein colocalizes with mitochondria in human hepatocytes and mouse liver (55), while the GCKIII kinases are confined to hepatocellular lipid droplets (16, 17, 19, 79). Lipid droplets are known to be tethered to mitochondria via integral membrane protein complexes and fusion of the outer membrane leaflets (80). It is therefore possible that MAP4K4 and the GCKIII kinases interact at the mitochondria-lipid droplet contact sites, and thereby mediate hepatocellular lipid homeostasis.

In addition to PDCD10 and MAP4K4, we also identified HSD17B11 as an interaction partner of several GCKIII kinases (i.e., MST3 and STK25) in the Y2H screens. We found that the silencing of HSD17B11 per se had no impact on lipid content in human hepatocytes. Remarkably, we discovered that the knockdown of MST3, STK25, or MST4 failed to reduce lipid accumulation in HSD17B11-deficient hepatocytes, indicating that the function of HSD17B11 is critical for the lipid-lowering effect of GCKIII inhibition. HSD17B11 belongs to the 17β-hydroxysteroid dehydrogenase family consisting of 15 members involved in steroid biosynthesis as well as lipid and retinol metabolism (54). HSD17B11 has been shown to associate with lipid droplets in Huh7 and VA-13 (human hepatoma cells) as well as HeLa cells, and overexpression of HSD17B11 increases the size of lipid droplets in VA-13 and HeLa cells, possibly through reduced adipose triglyceride lipase translocation to the lipid droplets (81, 82, 83). Interestingly, the hepatocellular function of another member of the 17β-hydroxysteroid dehydrogenase family, HSD17B13, has recently been thoroughly explored. HSD17B13 protein shares about 80% sequence similarity with HSD17B11 in the core catalytic domain (84). HSD17B13 primarily localizes to hepatic lipid droplets and genetic polymorphism in the *HSD17B13* gene results in loss of enzymatic activity and is linked to protection against MASH (85). However, the mechanisms by which genetic variants in *HSD17B13* mitigate MASH development are unknown and available data are conflicting. Neither overexpression nor knockdown of HSD17B13 in HepG2 (human hepatoma cells) was shown to impact lipid storage (86). In mice, hepatic overexpression of human *HSD17B13* increases liver TAG and cholesterol levels (87), while abrogation of the mouse *Hsd17b13* gene was reported to both promote hepatic steatosis and inflammation (88) or to have limited effects on MASH (89). Of note, three liver-specific oligonucleotide-based therapies targeting *HSD17B13* are currently being evaluated in clinical trials for the management of MASH (14). Although HSD17B13 was present in the primary human hepatocyte library used in the Y2H screens, it was not detected to interact with any of the GCKIII kinases. Together, this suggests that HSD17B13 regulates hepatocyte lipid metabolism via actions that do not involve the GCKIII kinases, whereas HSD17B11 is required for the GCKIII kinases to control lipid homeostasis.

There are some limitations associated with this study. Primarily, all experiments exploring the interplay between MST3, STK25, and MST4 were conducted using immortalized human cell lines, which may not fully mimic in vivo conditions. Consequently, additional research using mouse models, but also primary human cells, is essential to gain further insights into the shared mode of action of the GCKIII kinases. Moreover, in this investigation, we limited the evaluation of the lipotoxic effect of MST3, STK25, and MST4 to hepatocytes, macrophages, and hepatic stellate cells. However, given the complexity of MASLD pathophysiology, it is important to expand the analysis to additional cell types, including neutrophils, lymphocytes, dendritic cells, and liver sinusoidal endothelial cells, which are also known to contribute to MASLD development (90, 91, 92) and express high levels of both the GCKIII kinases and their key interaction partners (i.e., PDCD10, MAP4K4, and HSD17B11) https://www.livercellatlas.org/index.php, http://www.liverproteome.org/. Finally, this study sought to clarify the molecular network surrounding MST3, STK25, and MST4, yet comprehensive experimental validation of the interaction partners and phosphorylation targets of the GCKIII kinases identified by Y2H screens and in vitro kinase assays, respectively, is still pending, and will be the focus of our future studies.

In conclusion, we show that the members of the GCKIII kinase subfamily regulate hepatocyte lipid metabolism via common pathways. The results from a series of comprehensive experiments provide new insights into the role of MST3, STK25, and MST4, as well as their interactions with PDCD10, MAP4K4, and HSD17B11, in the control of liver lipid homeostasis. This knowledge can lay a platform for future investigations aimed at identifying novel targets for the prevention and/or treatment of MASLD and MASH, which remain major clinical challenges.

## Data availability

The data that support the findings of this study are available on request from the corresponding author.

## Conflicts of interest

The authors declare that they have no conflicts of interest with the contents of this article.

## Supplementary data

Supplematary information
