# Supplementary material for: GCKIII kinases control hepatocellular lipid homeostasis via shared mode of action
Source: J Lipid Res. 2024 Oct 10;65(11):100669. doi: 10.1016/j.jlr.2024.100669 (PMC11602991; doi:10.1016/j.jlr.2024.100669)
Supplement: Supplematary information [file mmc1.pdf]

Supplementary information

GCKIII Kinases Control Hepatocellular Lipid Homeostasis via Shared Mode of Action

Cansby et al.

Supplementary Figure S1

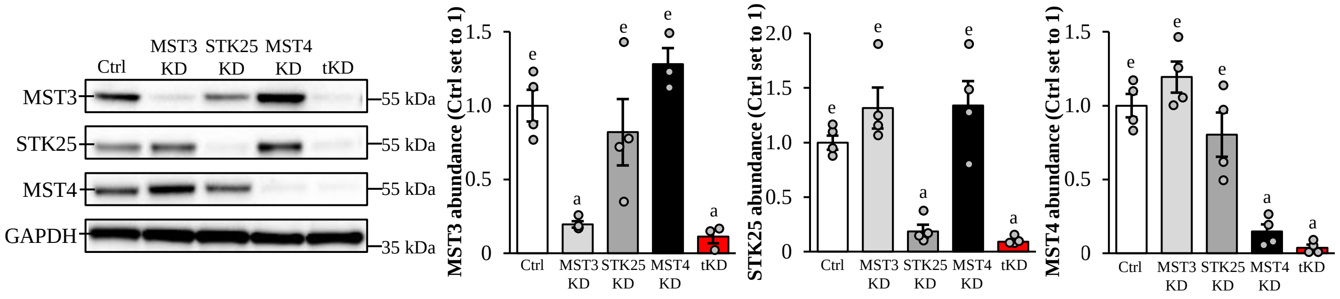

Supplementary Figure S1. Protein levels corresponding to the transfections presented in Fig. 1*B-G*, Supplementary Fig. S2, and Supplementary Fig. S3*A*. IHHs were transfected with *MST3*, *STK25*, *MST4*, and/or NTC siRNA, and incubated with oleic and palmitic acid for 48 h post-transfection. Cell lysates were analyzed by Western blot using antibodies specific for MST3, STK25, or MST4. Protein levels were quantified by densitometry; representative Western blots are shown with GAPDH used as a loading control. Data are mean ± SEM from 3-4 cell culture wells per group. Ctrl, control; KD, knockdown; tKD, triple knockdown. $^{a}P<0.05$ *versus* control; $^{e}P<0.05$ *versus* tKD

Supplementary Figure S2

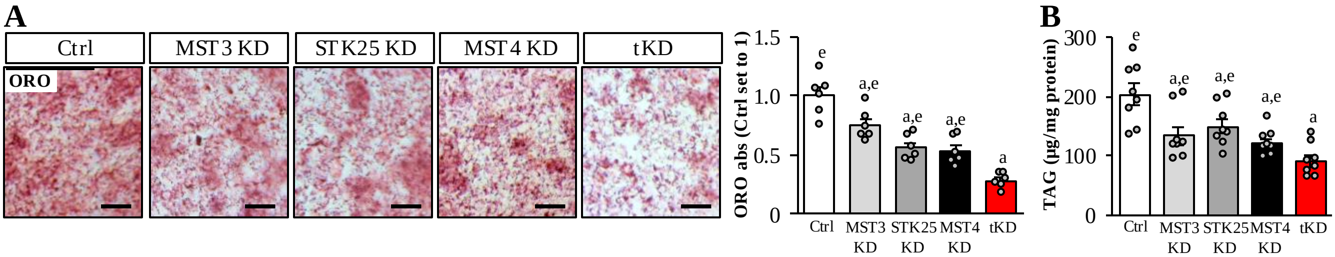

Supplementary Figure S2. Single knockdown of MST3, STK25, or MST4 suppresses fatty acid-induced lipid storage in human hepatocytes to a similar degree, with the decrease in ectopic fat accumulation being more pronounced in triple-deficient hepatocytes. IHHs were transfected with *MST3*, *STK25*, *MST4*, and/or NTC siRNA, and incubated with oleic and palmitic acid for 48 h post-transfection. (*A*) Representative images of cells stained with ORO. Quantification of the staining. Scale bar: 50 µm. (*B*) Colorimetric measurement of TAG content in cell lysates. Data are mean ± SEM from 6-8 cell culture wells per group. abs, absorbance; Ctrl, control; KD, knockdown; tKD, triple knockdown. $^{a}P<0.05$ *versus* control; $^{e}P<0.05$ *versus* tKD

Supplementary Figure S3

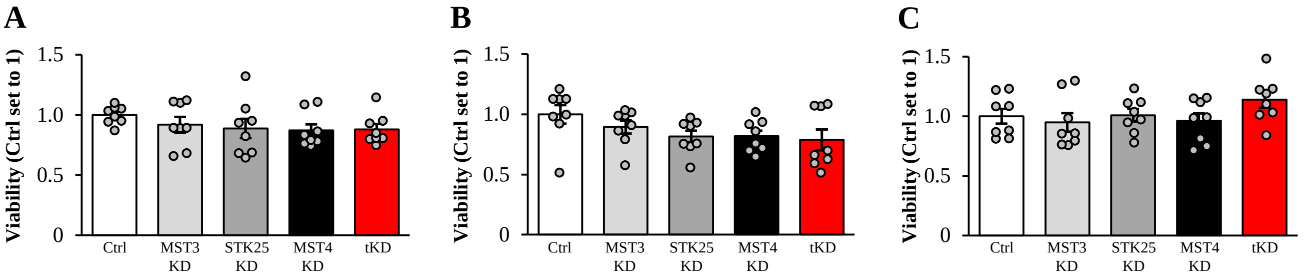

Supplementary Figure S3. Knockdown of MST3, STK25, and/or MST4 has no effect on cell viability. IHHs (*A*), THP-1-derived human macrophages (*B*), and LX-2 human hepatic stellate cells (*C*) were transfected with *MST3*, *STK25*, *MST4*, and/or NTC siRNA, and incubated with oleic and palmitic acid for 48 h post-transfection. Cell viability was assessed using resazurin. Data are mean ± SEM from 8 cell culture wells per group. Ctrl, control; KD, knockdown; tKD, triple knockdown.

Supplementary Figure S4

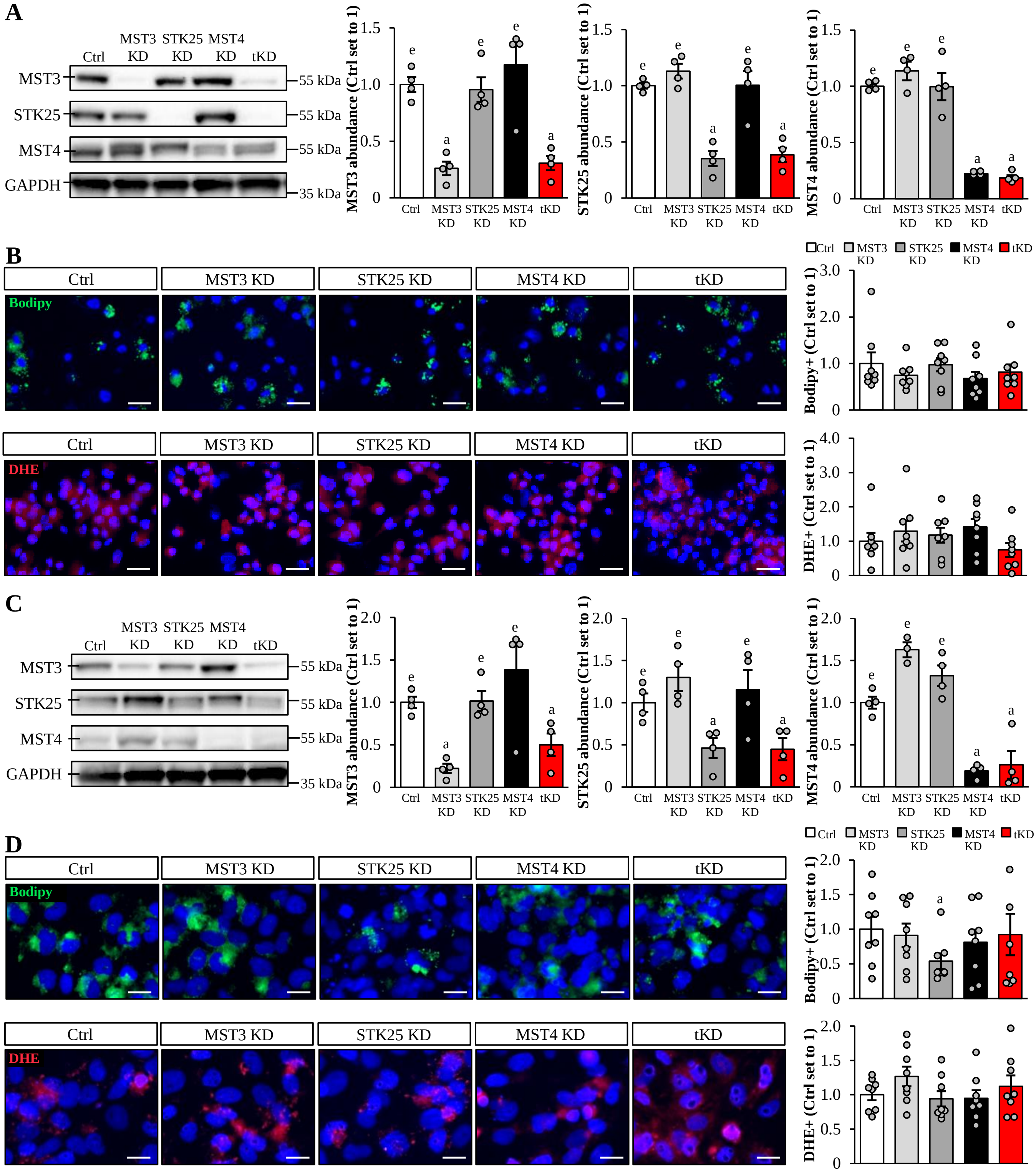

Supplementary Figure S4. Silencing of MST3, STK25, and/or MST4 has no impact on lipotoxicity in liver non-parenchymal cells. Cells were transfected with *MST3*, *STK25*, *MST4*, and/or NTC siRNA, and incubated with oleic and palmitic acid for 48 h post-transfection. (*A*,*C*) Cell lysates from THP-1-derived human macrophages (*A*) and LX-2 human hepatic stellate cells (*C*) were analyzed by Western blot using antibodies specific for MST3, STK25, or MST4. Protein levels were quantified by densitometry; representative Western blots are shown with GAPDH used as a loading control. (*B*,*D*) Representative images of THP-1-derived human macrophages (*B*) and LX-2 human hepatic stellate cells (*D*) stained with Bodipy (green) or DHE (red); nuclei stained with DAPI (blue). Quantification of the staining. Scale bar: 25 µm. Data are mean ± SEM from 4 (*A*,*C*) or 8 (*B*,*D*) cell culture wells per group. Ctrl, control; KD, knockdown; tKD, triple knockdown. [a]*P*<0.05 *versus* control; [e]*P*<0.05 *versus* tKD

Supplementary Figure S5

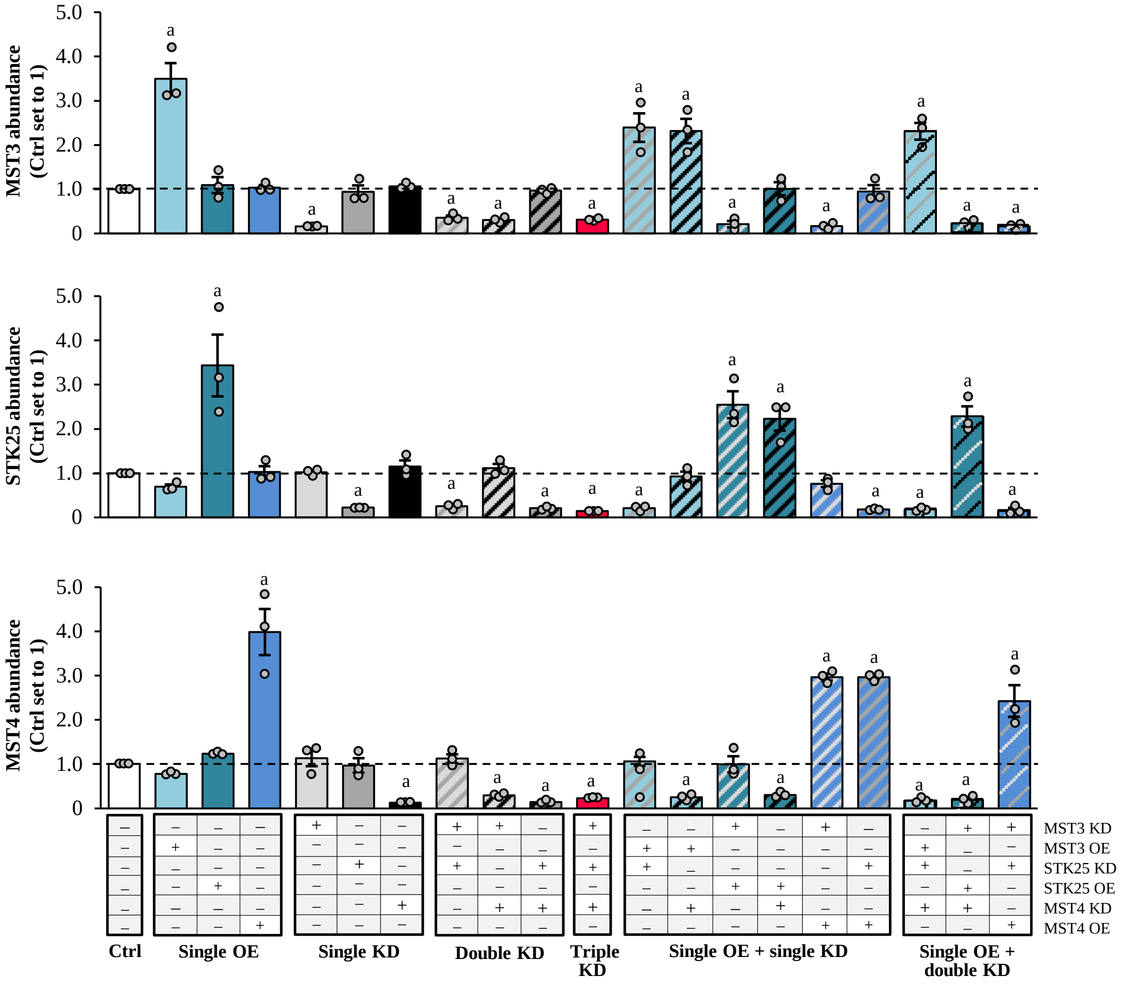

Supplementary Figure S5. Protein levels corresponding to Western blots presented in Fig. 2*A*. IHHs were transfected with different combinations of *MST3*, *STK25*, *MST4* siRNA, and/or with *MYC-MST3*, *FLAG-STK25*, *MYC-MST4* expression plasmids as indicated. Control cells were transfected with NTC siRNA and/or empty control plasmids. All cells were incubated with oleic and palmitic acid for 48 h post-transfection. Cell lysates were analyzed by Western blot using antibodies specific for MST3, STK25, or MST4. Protein levels were quantified by densitometry. Data are mean ± SEM from 3 cell culture wells per group. Ctrl, control; KD, knockdown; OE, overexpression. [a]*P*<0.05 *versus* control

Supplementary Figure S6

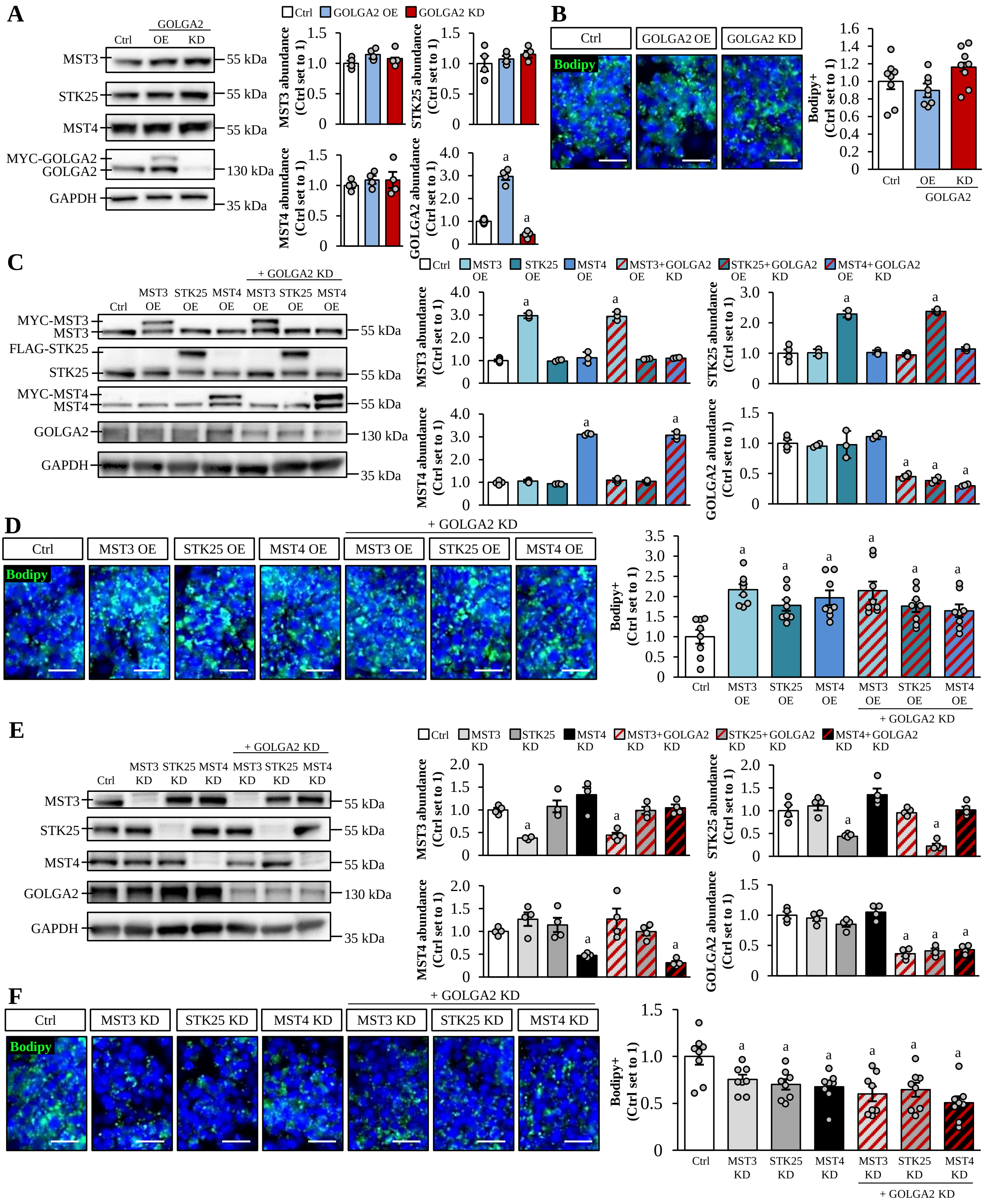

Supplementary Figure S6. Modifying the abundance of GOLGA2 does not affect hepatocellular lipid accumulation. IHHs were transfected with different combinations of *MST3*, *STK25*, *MST4*, *GOLGA2* siRNA, and/or with *MYC-MST3*, *FLAG-STK25*, *MYC-MST4*, *MYC-GOLGA2* expression plasmids as indicated. Control cells were transfected with NTC siRNA and/or empty control plasmids. All cells were incubated with oleic and palmitic acid for 48 h post-transfection. (*A,C,E*) Cell lysates were analyzed by Western blot using antibodies specific for MST3, STK25, MST4, or GOLGA2. Protein levels were quantified by densitometry; representative Western blots are shown with GAPDH used as a loading control. (*B,D,F*) Representative images of cells stained with Bodipy (green); nuclei stained with DAPI (blue). Quantification of the staining. Scale bar: 30 µm. Data are mean ± SEM from 3-4 (*A,C,E*) or 8 (*B,D,F*) cell culture wells per group. For (*E-F*), representative results from 2 independent experiments are shown. Ctrl, control; KD, knockdown; OE, overexpression. [a]*P*<0.05 *versus* control

Supplementary Figure S7

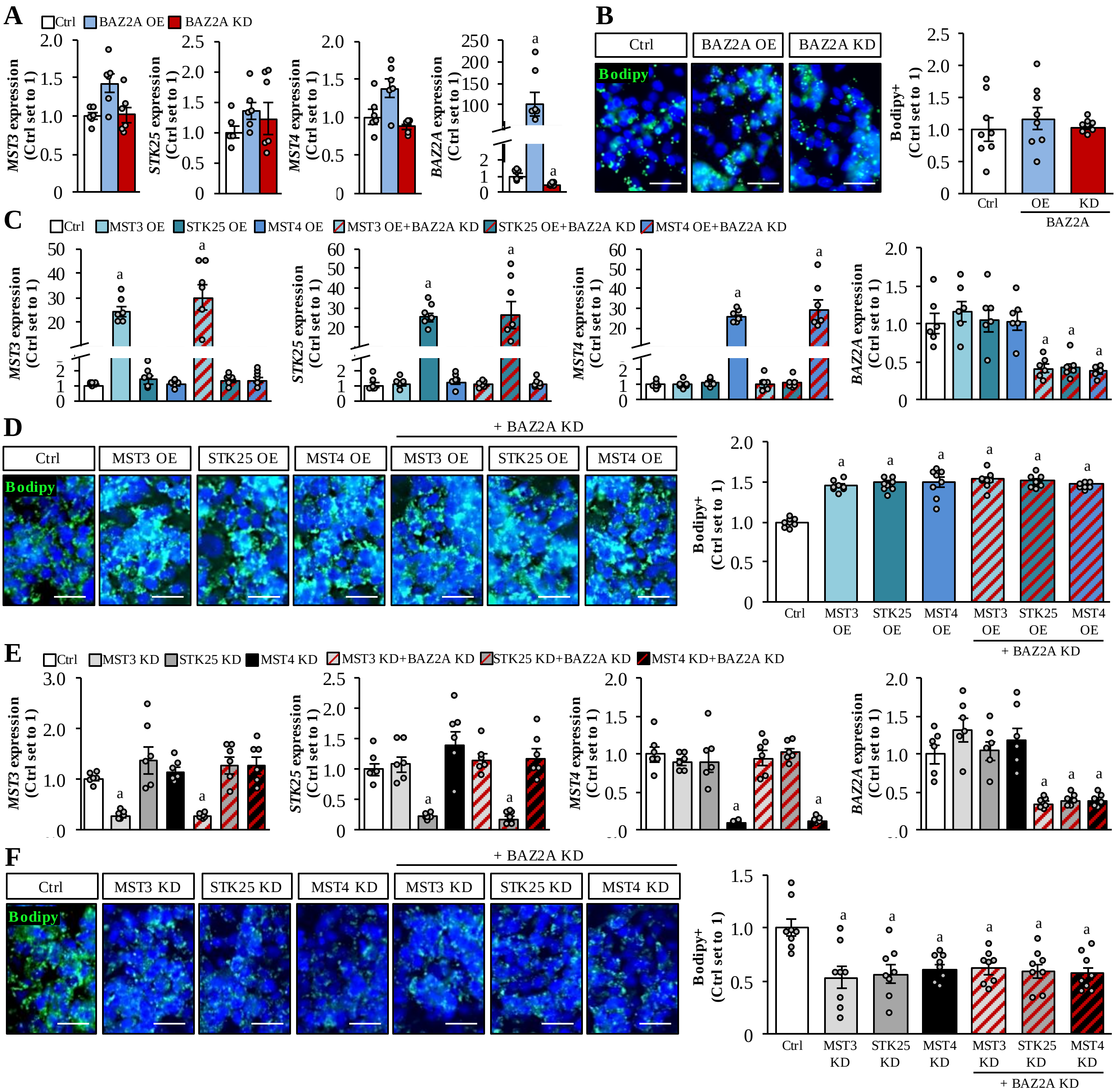

Supplementary Figure S7. Modifying the abundance of BAZ2A does not affect hepatocellular lipid accumulation. IHHs were transfected with different combinations of *MST3*, *STK25*, *MST4*, *BAZ2A* siRNA, and/or with *MYC-MST3*, *FLAG-STK25*, *MYC-MST4*, *MYC-BAZ2A* expression plasmids as indicated. Control cells were transfected with NTC siRNA and/or empty control plasmids. All cells were incubated with oleic and palmitic acid for 48 h post-transfection. (*A*,*C*,*E*) *MST3*, *STK25*, *MST4*, and *BAZ2A* mRNA expression assessed by qRT-PCR. (*B*,*D*,*F*) Representative images of cells stained with Bodipy (green); nuclei stained with DAPI (blue). Quantification of the staining. Scale bar: 30 µm. Data are mean ± SEM from 6-8 cell culture wells per group. For (*A*-*B*), representative results from 2 independent experiments are shown. Ctrl, control; KD, knockdown; OE, overexpression. [a]*P*<0.05 *versus* control

Supplementary Figure S8

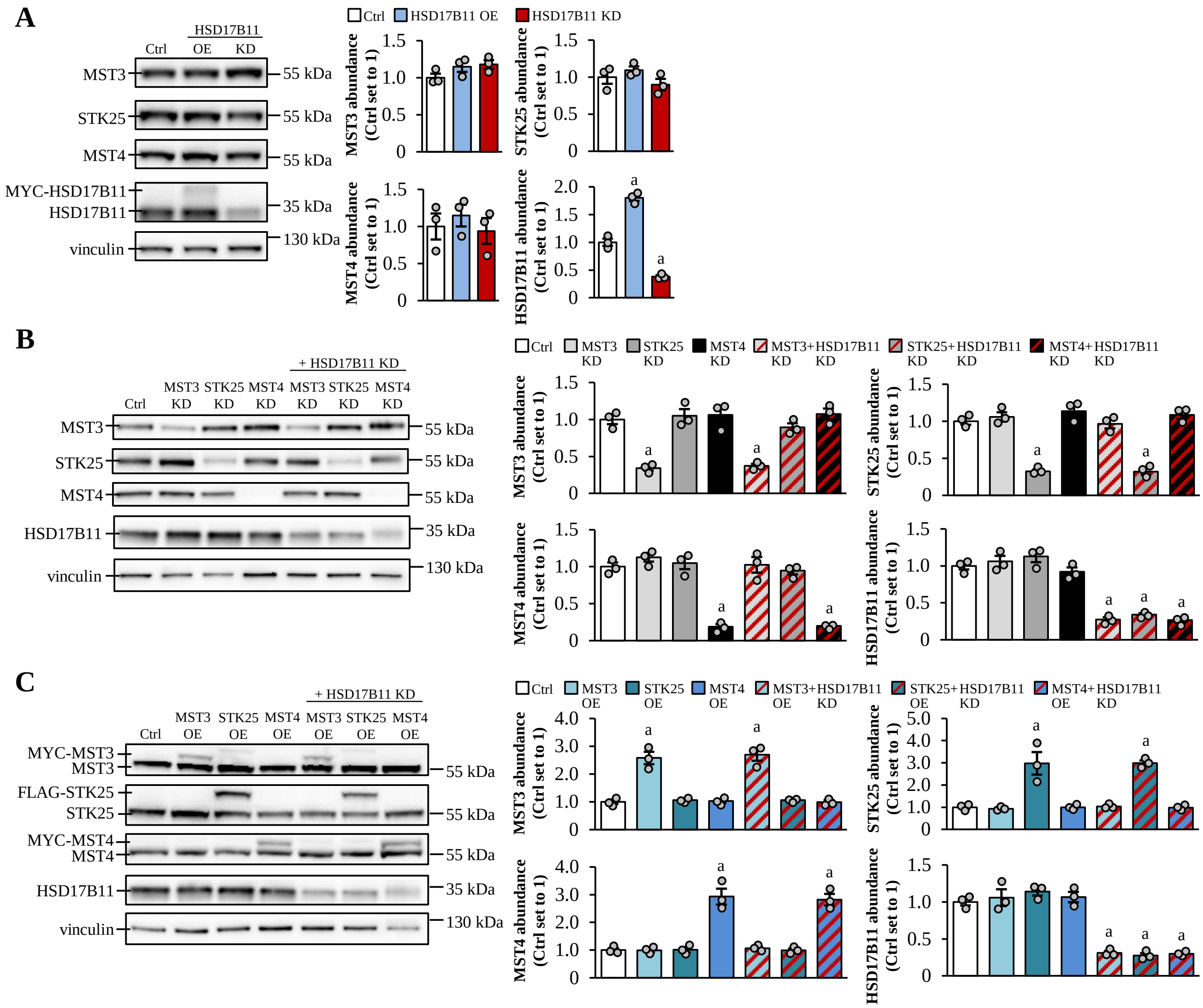

Supplementary Figure S8. Protein levels corresponding to the transfections presented in Fig. 5*A-C* and Supplementary Fig. S9. IHHs were transfected with different combinations of *MST3, STK25, MST4, HSD17B11* siRNA, and/or with *MYC-MST3, FLAG-STK25, MYC-MST4, MYC-HSD17B11* expression plasmids as indicated. Control cells were transfected with NTC siRNA and/or empty control plasmids. All cells were incubated with oleic and palmitic acid for 48 h post-transfection. Cell lysates were analyzed by Western blot using antibodies specific for MST3, STK25, MST4, or HSD17B11. Protein levels were quantified by densitometry; representative Western blots are shown with vinculin used as a loading control. Data are mean ± SEM from 3 cell culture wells per group. Representative results from 2-3 independent experiments are shown. Ctrl, control; KD, knockdown; OE, overexpression. [a]*P*<0.05 *versus* control

Supplementary Figure S9

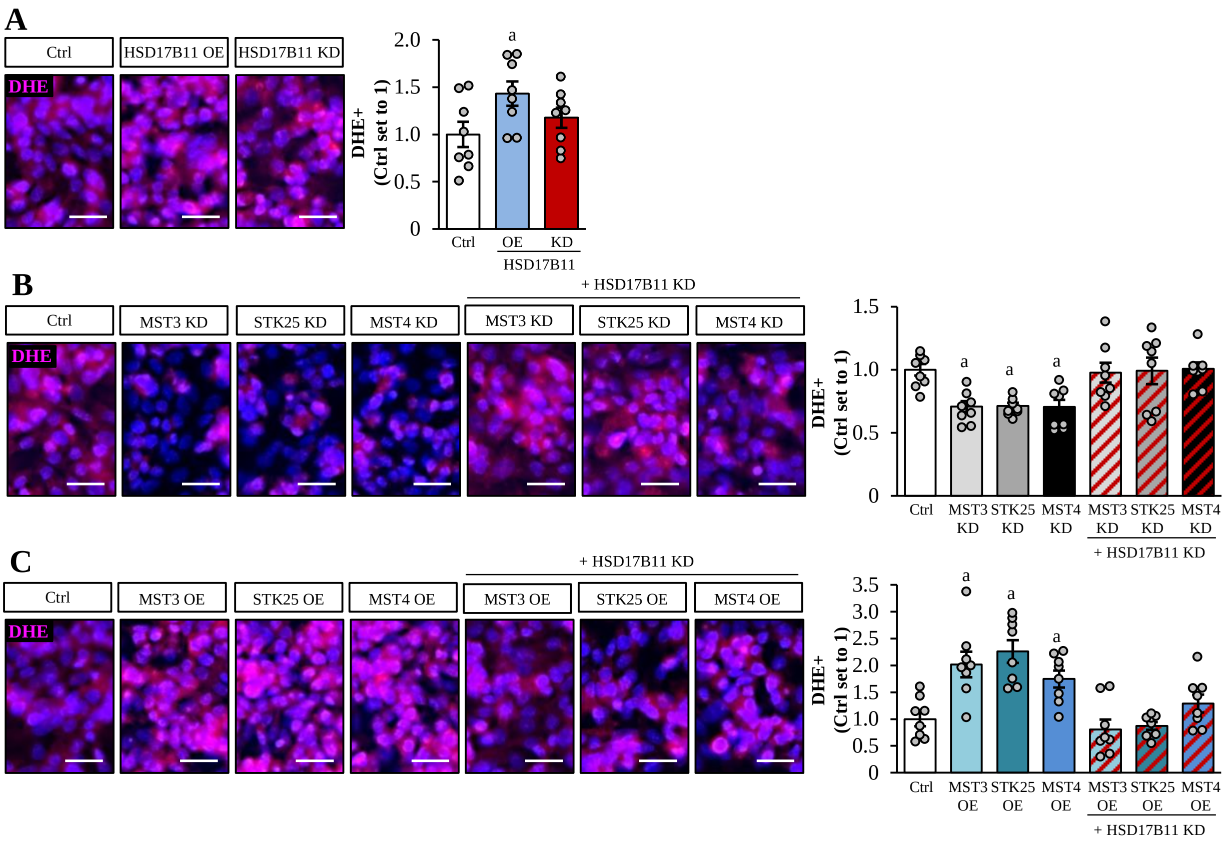

Supplementary Figure S9. Regulation of hepatocellular oxidative stress by GCKIII kinases is mediated by HSD17B11. IHHs were transfected with different combinations of *MST3*, *STK25*, *MST4*, *HSD17B11* siRNA, and/or with *MYC-MST3*, *FLAG-STK25*, *MYC-MST4*, *MYC-HSD17B11* expression plasmids as indicated. Control cells were transfected with NTC siRNA and/or empty control plasmids. All cells were incubated with oleic and palmitic acid for 48 h post-transfection. Representative images of cells stained with DHE (pink); nuclei stained with DAPI (blue). Quantification of the staining. Scale bar: 30 µm. Data are mean ± SEM from 8 cell culture wells per group. Ctrl, control; KD, knockdown; OE, overexpression. [a]$P<0.05$ *versus* control

Supplementary Figure S10

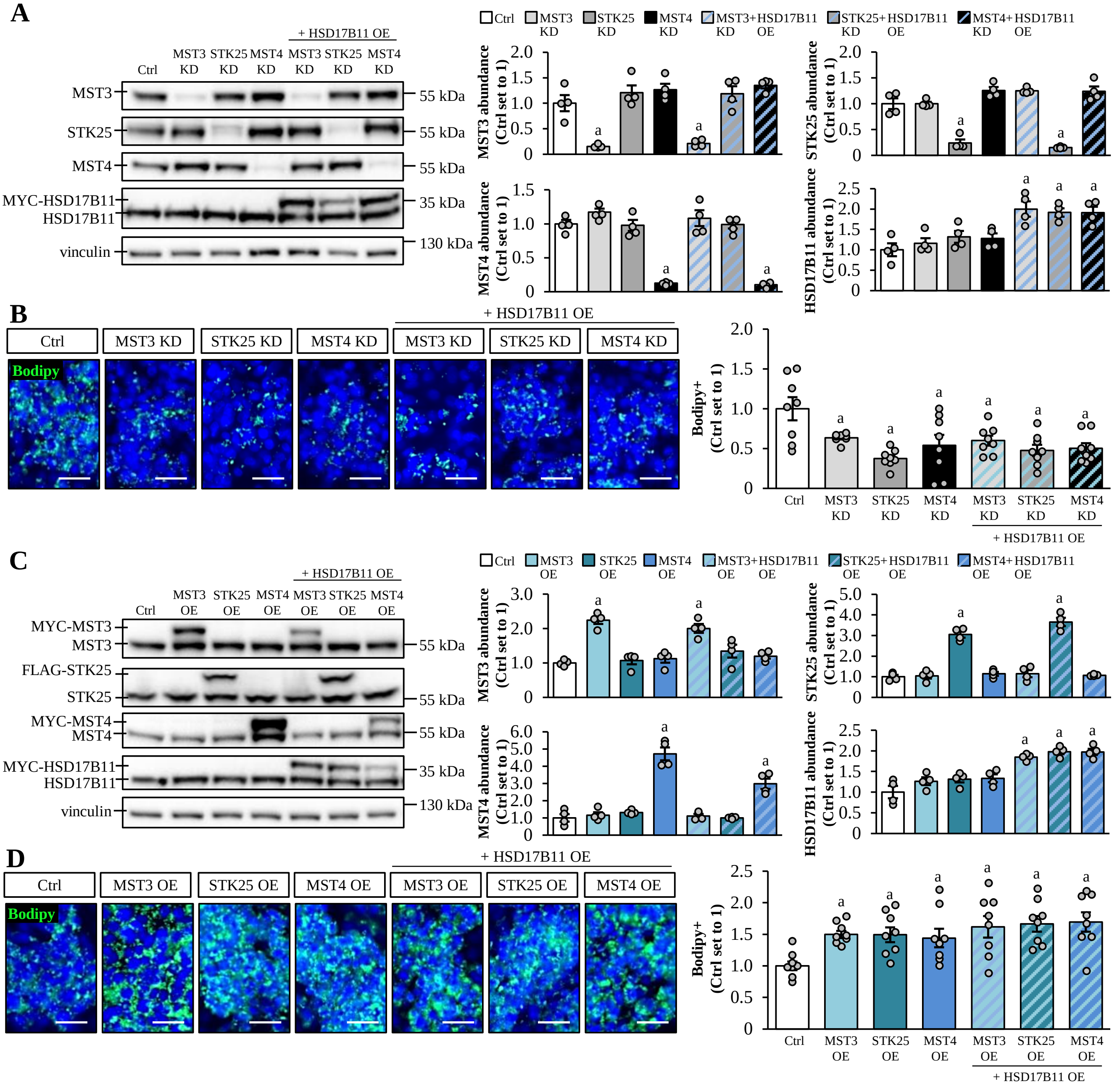

Supplementary Figure S10. Increasing the abundance of HSD17B11 does not affect the lipid content-modifying activity of the GCKIII kinases. IHHs were transfected with different combinations of *MST3*, *STK25*, *MST4* siRNA, and/or with *MYC-MST3*, *FLAG-STK25*, *MYC-MST4*, *MYC-HSD17B11* expression plasmids as indicated. Control cells were transfected with NTC siRNA and/or empty control plasmids. All cells were incubated with oleic and palmitic acid for 48 h post-transfection. (*A*,*C*) Cell lysates were analyzed by Western blot using antibodies specific for MST3, STK25, MST4, or HSD17B11. Protein levels were quantified by densitometry; representative Western blots are shown with vinculin used as a loading control. (*B*,*D*) Representative images of cells stained with Bodipy (green); nuclei stained with DAPI (blue). Quantification of the staining. Scale bar: 30 µm. Data are mean ± SEM from 4 (*A*,*C*) or 8 (*B*,*D*) cell culture wells per group. Representative results from 2 independent experiments are shown. Ctrl, control; KD, knockdown; OE, overexpression. [a]*P*<0.05 *versus* control

Supplementary Figure S11

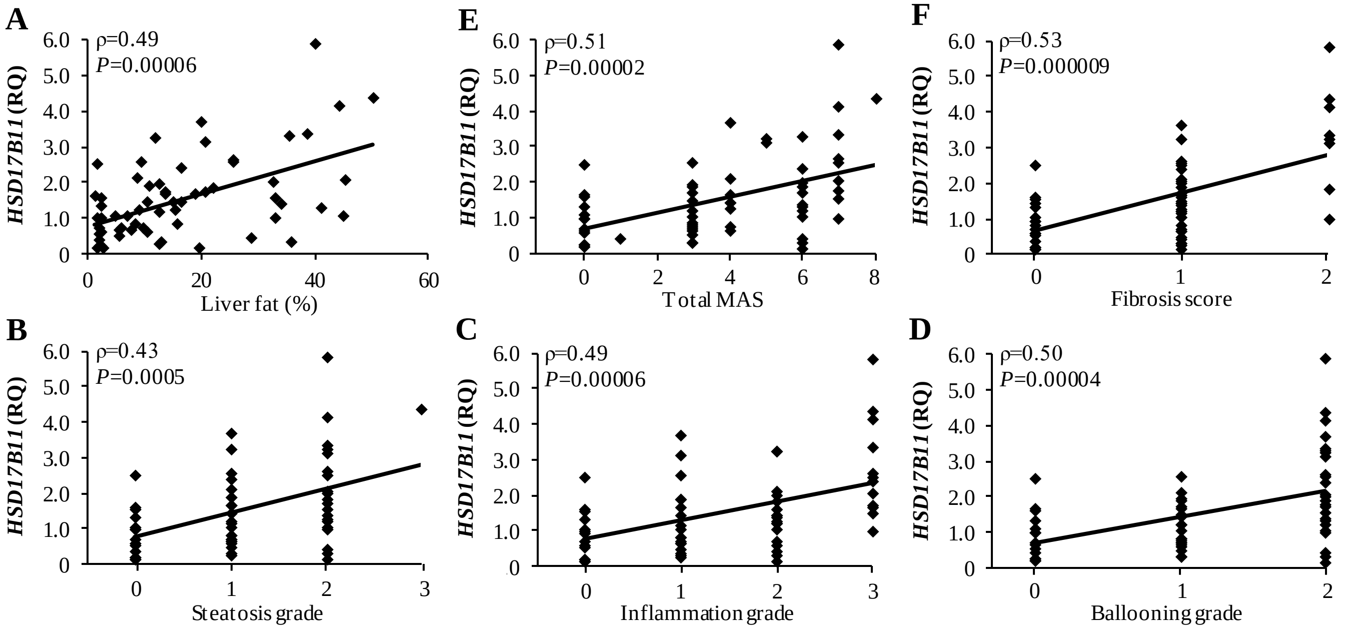

Supplementary Figure S11. Hepatic *HSD17B11* expression is significantly and positively correlated with the severity of MASLD. (*A*) Correlation between hepatic *HSD17B11* mRNA expression and liver fat content measured by magnetic resonance spectroscopy (^{1}H-MRS). (*B*-*F*) Correlation between *HSD17B11* mRNA abundance determined in human liver biopsies by qRT-PCR and the severity of the individual components of MAS (liver steatosis, inflammation, hepatocellular ballooning; *B*-*D*) as well as composite MAS (*E*), and the histological score of liver fibrosis (*F*). n=62. RQ, relative quantification.

Supplementary Figure S12

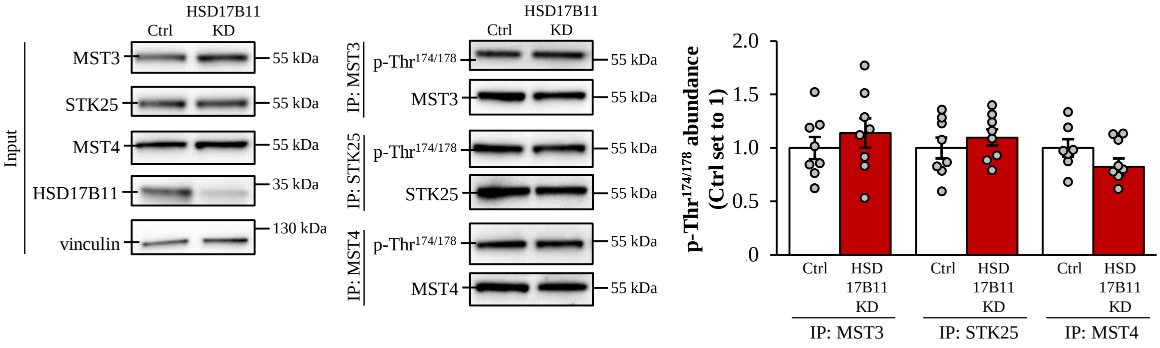

Supplementary Figure S12. HSD17B11 does not affect the phosphorylation level of the GCKIII kinases in hepatocytes. IHHs were transfected with *HSD17B11* siRNA or NTC siRNA and incubated with oleic and palmitic acid for 48 h post-transfection. Cell lysates were immunoprecipitated with Dynabeads Protein G magnetic beads and subjected to Western blot using antibodies specific for MST3, STK25, MST4, HSD17B11, or phosohpo-MST3/-STK25/-MST4 ($Thr^{174/178}$). Protein levels were quantified by densitometry; representative Western blots are shown with vinculin used as a loading control. Data are mean ± SEM from 8 cell culture wells per group. Ctrl, control; IP, immunoprecipitation; KD, knockdown.

Supplementary Figure S13

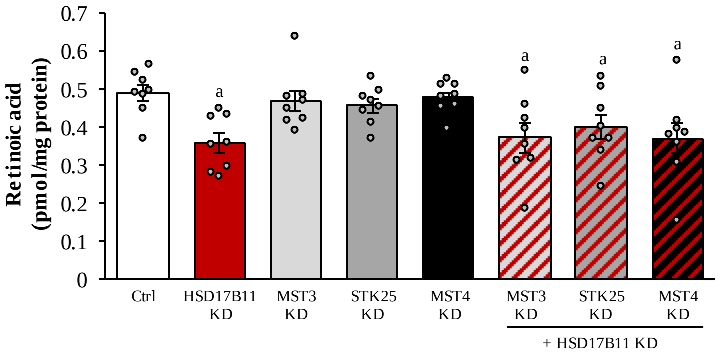

Supplementary Figure S13. Silencing of MST3, STK25, or MST4 does not change the enzymatic activity of HSD17B11. IHHs were transfected with different combinations of *MST3*, *STK25*, *MST4*, *HSD17B11*, and/or NTC siRNA, and incubated with oleic and palmitic acid for 48 h post-transfection. To measure retinol dehydrogenase activity, cells were incubated with all-trans-retinol and retinoic acid concentration was then analyzed in cell lysates. Data are mean ± SEM from 8 cell culture wells per group. Ctrl, control; KD, knockdown. [a]$P<0.05$ *versus* control

Supplementary Table S1. List of antibodies used for Western blot and immunofluorescence analysis

Type	Antibody name and catalogue number	Working dilution	Company
Primary antibody	anti-MST3 (#3723)	1:1000	Cell Signaling Technology (Boston, MA)
	anti-STK25 (25821-1-AP)	1:1000	Proteintech (Chicago, IL)
	anti-MST4 (#3822)	1:1000	Cell Signaling Technology
	anti-4-HNE (ab46545)	1:500	Abcam (Cambridge, UK)
	anti-8-oxoG (ab62623)	1:500	Abcam
	anti-CHOP (MA1-250)	1:250	Invitrogen (Waltman, MA)
	anti-KDEL (ab176333)	1:500	Abcam
	anti-PDCD10 (ab180706)	1:1000	Abcam
	anti-MAP4K4 (#3485)	1:1000	Cell Signaling Technology
	anti-MST4+MST3+STK25 (phospho-Thr) (orb185140)	1:1000	Biorbyt (Cambridge, UK)
	anti-GOLGA2 (PA1-077)	1:1000	Invitrogen
	anti-HSD17B11 (PA5-54469)	1:1000	Invitrogen
	anti-GAPDH (sc-47724)	1:1000	Santa Cruz Biotechnology (Santa Cruz, CA)
	anti-vinculin (sc-7269)	1:1000	Santa Cruz Biotechnology
Secondary antibody	Alexa Fluor-488-labeled anti-mouse IgG (A21202)	1:500	Invitrogen
	Alexa Fluor-488-labeled anti-rabbit IgG (A11008)	1:500	Invitrogen
	Alexa Fluor-594-labeled anti-mouse IgG (A11005)	1:500	Invitrogen
	Alexa Fluor-594-labeled anti-rabbit IgG (A21207)	1:500	Invitrogen
	anti-rabbit IgG (#7074)	1:1000	Cell Signaling Technology
	anti-mouse IgG (#7076)	1:1000	Cell Signaling Technology

Supplementary Table S2. Characteristics of the study participants

	MASLD score steatosis*			
	**0**	**1**	**2**	**3**
N (women/ men)	16 (5/11)	28 (14/14)	20 (10/10)	2 (0/2)
BMI (kg/m²)	24.8±1.3	35.2±5.9§§	33.2±5.0§§	37.1±12
Body fat (%)	24.1±4.3	39.7±9.1§§	32.5±8.3§§	37.3±7.8
Liver fat (%)	2.2±0.8	11.5±3.9§§	31.7±9.2$^{§§,\#\#}$	50.0
MASLD score ballooning*	0	1.2±0.4§§	2$^{§§,\#\#}$	2
MASLD score lobular inflammation*	0.1±0.3	1.5±0.6§§	2.4±0.6$^{§§,\#\#}$	2.5±0.7
MASLD score fibrosis*	0	0.4±0.4§	2.0±0.9$^{§§,\#\#}$	2
Visceral fat area (cm²)	59±33	162±31§§	270±43$^{§§,\#\#}$	384±44
Subcutaneous fat area (cm²)	114±231	922±251§§	476±258$^{§§,\#}$	564±251
Waist to hip ratio	0.9±0.1	1.1±0.2§	1.1±0.1§	1.1±0.1
FPG (mmol/l)	5.7±0.9	5.7±0.9	5.7±0.8	5.5±0.7
FPI (pmol/l)	80±127	205±147§§	198±122§§	135±58
HbA1c (%)	5.6±0.5	5.8±0.4	5.9±0.6	5.7±0.1
Total Cholesterol (mmol/l)	4.6±0.7	5.2±0.8§	5.7±0.9$^{§§,\#\#}$	6.5±0.75
HDL-Cholesterol (mmol/l)	1.4±0.4	1.3±0.3	1.3±0.2	1.5±0.1
LDL-Cholesterol (mmol/l)	2.9±0.8	2.9±0.8	3.3±0.9$^{§,\#}$	4.3±1.3
Triglycerides (mmol/l)	1.2±0.7	1.9±0.7§§	2.3±0.6$^{§§,\#}$	2.8±1.3
ALAT (µkat/l)	0.5±0.2	0.5±0.2	0.6±0.2§	0.9±0.3
ASAT (µkat/l)	0.4±0.1	0.5±0.09	0.6±0.2§	0.9±0.4
GGT (µkat/l)	0.5±0.2	0.6±0.2	0.6±0.3	0.7±0.5
Adiponectin (µg/ml)	9.1±3.9	5.0±2.9§	3.7±2.5§§	4.2±0.7
Leptin (pg/ml)	7.5±12.4	25±13.6§§	29.6±17§§	19.4±3.6

*histological features were evaluated in liver sections as recommended by the MASH Clinical Research Network (1).
Data are mean ± SD. ALAT; alanine-aminotransferase; ASAT, aspartate-aminotransferase; BMI, body mass index; FPG, fasting plasma glucose; FPI, fasting plasma insulin; GGT, gamma-glutamyl transpeptidase; HbA1c, glycated haemoglobin; HDL, high density lipoprotein; LDL, low density lipoprotein. $^{§}P<0.05$; $^{§§}P<0.01$ *versus* the MASLD score steatosis 0 group; $^{\#}P<0.05$, $^{\#\#}P<0.01$ *versus* the MASLD score steatosis 1 group. For the MASLD score steatosis 3 group, statistical analysis was omitted because of the low number of patients in this group

References

1. Kleiner DE, Brunt EM, Van Natta M, Behling C, Contos MJ, Cummings OW, Ferrell LD, et al. Design and validation of a histological scoring system for nonalcoholic fatty liver disease. Hepatology 2005;41:1313-1321.

Supplementary Table S3. Candidate interactors of MST3 detected in a Y2H screen using MST3 as bait

PBS	Accession number	Symbol	Name	Aliases	Number of hits	Start	Stop	Function	Identified on liver lipid droplets
B	Q9UIF9	*BAZ2A*[#]	Bromodomain adjacent to zinc finger domain protein 2A		1<br>1<br>1	ND<br>2769<br>2775	4006<br>4006<br>3090	BAZ2A is a subunit within the nuclear remodeling complex (NoRC), that recruits the NoRC to ribosomal RNA genes, leading to their transcriptional repression.	No
B	Q9H6S3	*EPS8L2*	EPS8-like protein 2	EPS8R2	1<br>1<br>1	330<br>531<br>659	912<br>873<br>943	EPS8P2, a substrate for the epidermal growth factor receptor (EGFR) kinase, contributes to mitogenesis and tumorigenesis through the activation of downstream signalling pathways facilitated by EGFR.	Yes (1–3)
C	O14595	*CTDSP2*	CTD small phosphatase 2	SCP2; OS4	1<br>1	261<br>282	516<br>533	CTDSP2 (EC 3.1.3.16) is a phosphatase that catalyzes the dephosphorylation of the C-terminal domain of RNA polymerase II, resulting in gene silencing by repressing RNA transcription.	No
D	P00736	*C1R*	Complement component C1r		1	681	2030	C1R (EC 3.4.21.41) is a subunit in a complex that controls the first enzymatic event in the cascade of the classical pathway of the complement system.	No
D	P01024	*C3*	Complement component C3	ASP	1<br>4	3387<br>3387	ND<br>3929	C3 is a crucial effector component of the complement system.	Yes (1,2)
D	Q00403	*GTF2B*	General transcription factor IIB	TFIIB	1<br>1	-46<br>-46	163<br>963	GTF2B is a general transcription factor that is involved in the formation of the RNA polymerase II preinitiation complex and aids in stimulating transcription initiation.	Yes (1)
D	O94953	*KDM4B*	Lysine demethylase 4B	JMJD2B; KIAA0876	2	1815	2146	KDM4B (EC 1.14.11) is a histone demethylase with catalytic activity against histone modification, preventing chromatin compaction and gene repression.	No
D	Q9Y6X3	*MAU2*	MAU2 sister chromatid cohesion factor	SCC4; KIAA0892	1	-16	919	MAU2 is a component of the loader complex that facilitates the initial association of cohesin and DNA during telophase, thus ensuring genomic stability.	No
D	P42695	*NCAPD3*	Non-SMC condensin II complex subunit D3	CAPD3; KIAA0056	1	1485	ND	NCAPD3 is one of three non-structural maintenance of chromosomes (non-SMC) proteins that characterize condensin-2 complex; it is required for chromosomal condensation in prophase.	No
D	P04085	*PDGFA*	Platelet-derived growth factor, alpha polypeptide		1	-217	187	PDGFA is a subunit of platelet-derived growth factor (PDGF), that plays an essential role in the regulation of embryonic development, cell proliferation and migration, survival, and chemotaxis.	No
D	Q9GZM3	*POLR2J2*	RNA polymerase II subunit J2		1	63	496	POLR2J2, a variant subunit of RNA polymerase II, interacts with Special AT-Rich Sequence Binding Protein 1 (SATB1) to regulate chromatin-loop architecture and transcription.	No
D	Q92954	*PRG4*	Proteoglycan 4	MSF; SZP; CACP	1	2871	3932	PRG4 is a multifunctional mucin-like glycoprotein that plays a pivotal role in maintaining the lubricated surfaces of articular cartilage but also acts as a signalling molecule in the regulation of inflammation.	Yes (1)

PBS	Accession number	Symbol	Name	Aliases	Number of hits	Start	Stop	Function	Identified on liver lipid droplets
E	O43143	*DHX15*	DHEA-box helicase 15	DDX15; DBP1; HRH2	1	738	ND	DHX15 (EC 3.6.4.13) is an ATP-dependent RNA helicase that functions as an RNA sensor, and participates in pre-mRNA splicing, ribosome biogenesis and assembly.	Yes (1,2,3)
N/A	P61158	*ACTR3*	Actin-related protein 3	ARP3	1<br>1	262<br>262	-217<br>-188	ACTR3 is an essential component of the actin-related protein complex (ARP2/3), involved in cell motility and the endothelial-mesenchymal transition process.	Yes (1)
N/A	Q7Z591	*AKNA*	AT-hook transcription factor AKNA		1<br>1	3334<br>3334	ND<br>3034	AKNA is a microtubule organization protein; its DNA binding motif interacts with AT-rich promoters to regulate transcription of inflammatory genes.	No
N/A	P02768	*ALB*	Albumin		1	ND	63	ALB is a predominant plasma protein that plays a key role in preserving vascular endothelial integrity, regulating acid-base balance, modulating inflammation, and serving as a carrier for both endogenous and exogenous compounds.	Yes (2,1,4)
N/A	P59780	*AP3S2*	Adaptor-related protein complex 3 sigma-2 subunit		1	649	-4	AP3S2 is a part of the AP-3 complex, which interacts with coated profiles of endosomal tubules to regulate the homeostasis of plasma membrane receptors.	No
N/A	O75915	*ARL6IP5*	ADP-ribosylation factor-like GTPase 6-interacting protein 5	PRAF3; GTRAP3-18; JWA	1	328	-61	ARL6IP5 is an integral endoplasmic reticulum (ER) protein, which negatively regulates ER protein trafficking.	Yes (1)
N/A	Q9H1I8	*ASCC2*	Activating signal cointegrator 1 complex subunit 2	p100	1	ND	495	ASCC2 is a subunit of the human ribosomal-associated quality control complex, that interacts with K63-linked polyubiquitin chains to facilitate the dissociation of collided ribosomes.	Yes (1)
N/A	Q9NYV4	*CDK12*	Cyclin-dependent kinase 12	CRKRS; CRK7; KIAA0904	1	3102	2625	CDK12 (EC 2.7.11.23) is a cyclin-dependent kinase that regulates transcriptional elongation and RNA splicing, impacting the DNA damage and repair process.	No
N/A	Q7Z7K6	*CENPV*	Centromere protein V		1	ND	925	CENPV serves as bridging link that connects the chromosomal passenger complex (CPC), primary contraction of mitotic chromosomes, and peristomal heterochromatin.	Yes (1)
N/A	P07858	*CTSB*	Cathepsin B	CATB; APPS	1	ND	544	CTSB (EC 3.4.22.1) is a lysosomal protease integral to lysosome-related processing, including protein turnover, degradation, and cell-death.	Yes (1,2,4)
N/A	P11182	*DBT*	Dihydrolipoamide branched-chain transacylase	BCATE2	1	4057	3660	DBT (EC 2.3.1.168) is a component of branched-chain alpha-keto acid dehydrogenase complex that catalyses the conversion of alpha-keto acids to acyl-CoA.	Yes (1)
N/A	Q7Z478	*DHX29*	DExH-box helicase		1<br>1	-2094<br>-1824	-1392<br>-1558	DHX29 (EC 3.6.4.13) is an ATP-binding RNA helicase essential for scanning stable secondary structures and translation initiation of various viral mRNAs through its ribosome-stimulated nucleoside triphosphatase (NTPase) activity.	Yes (1)
N/A	O60610	*DIAPH1*	Diaphanous-related formin 1	DIA1	1	4062	4616	DIAPH1, a member of the formin protein family, acts as an effector of Rho small guanosine triphosphatases (GTPases) to control actin polymerization, cell polarity, shape, and motility, as well as vesicular trafficking.	Yes (1)

PBS	Accession number	Symbol	Name	Aliases	Number of hits	Start	Stop	Function	Identified on liver lipid droplets
N/A	P78344	*EIF4G2*	Eukaryotic translation initiation factor 4-gamma 2	p97; DAP5	2	32	809	EIF4G2 is a subunit of the scaffolding protein complex eukaryotic translation initiation factor 4F (EIF4F), which represses cap-dependent translation and promotes cap-independent translation initiation.	Yes (1,2,4)
N/A	P55899	*FCGRT*	Fc fragment of IgG receptor and transporter		1	319	496	FCGRT is related to the class 1 major histocompatibility complex and protects against catabolism of IgG and albumin in the liver to maintain protein homeostasis.	Yes (1)
N/A	P32455	*GBP1*	Guanylate-binding protein 1, interferon-inducible, 67-kD		1	460	-52	GBP1 (EC 3.6.1) is a member of the GTPase family crucial for the maturation of autophagosomes in response to pathogens; it also promotes inflammasome assembly and autophagy.	No
N/A	P49841	*GSK3B*	Glycogen synthase kinase 3-beta		1	-731	985	GSK3B (EC 2.7.11.1) is a protein kinase that negatively regulates glycogen synthesis in response to insulin and Wnt/β-catenin pathway stimulation.	Yes (1)
N/A	P13747	*HLA-E*	Major histocompatibility complex, class I, E	HLAE; QA1	1	1819	2437	HLA-E serves as a ligand for CD94-NKG2A receptor, acting as checkpoint in natural killer cell immune surveillance.	No
N/A	Q8NBQ5	*HSD17B11*[#]	17-beta-hydroxysteroid dehydrogenase XI	17-BETA-HSDXI; RETSDR2; PAN1B	1	1191	1546	HSD17B11 (EC 1.1.1.62) belongs to the short-chain dehydrogenase/reductase family, involved in sex steroid, lipid, and cholesterol metabolism; it is also reported to be associated with lipid droplet formation.	Yes (1,2,4)
N/A	Q6ZMZ0	*IBRDC3*	IBR domain-containing protein 3	NKLAM	1<br>2<br>1<br>2<br>1	ND<br>512<br>581<br>635<br>1579	1156<br>1010<br>797<br>788<br>1535	IBRDC3 (EC 6.3.2.19) is a E3 ubiquitin-protein ligase which selectively transfers ubiquitin from E2 conjugates to target substrates, facilitating the regulation of cellular processes.	No
N/A	P10914	*IRF1*	Interferon regulatory factor 1		1	ND	3005	IRF1 is a member of the interferon regulatory factor family, which induces interferon β to facilitate innate and adaptive immunity and also possesses an anti-oncogenic effect.	No
N/A	Q14624	*ITIH4*	Inter-alpha-trypsin inhibitor heavy chain 4	ITIHL1; IHRP; PK120	1	55	369	ITIH4, a member of the serine protease inhibitor family, possesses systematic anti-inflammatory properties and functions as an anti-apoptotic and matrix stabilizing molecule.	Yes (1)
N/A	P30414	*NKTR*	Natural killer cell triggering receptor		1	2185	2973	NKTR (EC 5.2.1.8) is ER foldase protein that catalyzes the isomerization of prolines to facilitate the folding of proteins involved in signalling transduction, apoptosis, and adaptive immunity pathways.	No
N/A	F8WF36	*PALM2AKAP2*	PALM2 and AKAPA fusion gene		1<br>1<br>2<br>2	ND<br>2247<br>2250<br>2253	1220<br>1193<br>1785<br>1220	PALM2AKAP2 is a fusion gene of PALM2 and AKAP2 with an unknown function.	No
N/A	Q9BUL8	*PDCD10*[###]	Programmed cell death 10	CCM3; TRAF15	3	25	760	PDCD10 is a component of striatin-interacting phosphatase and kinase (STRIPAK) complex involved in apoptosis regulation, cell migration, and Golgi assembly.	Yes (1)

PBS	Accession number	Symbol	Name	Aliases	Number of hits	Start	Stop	Function	Identified on liver lipid droplets
N/A	Q7L8J4	*SH3BP5L*	SH3 domain-binding protein 5-like	KIAA1720	1	-412	ND	SH3BP5L is a guanine nucleotide exchange factor required for the activation of Ras-related protein Rab-11A during lumenogenesis.	No
N/A	Q7Z392	*TRAPPC11*	Trafficking protein particle complex subunit 11	C4ORF41	2<br>1	487	820	TRAPPC11 is a component of the mammalian transport protein particle (TRAPP III) complex involved in intracellular vesicle trafficking in the early secretory pathway and assembly of the complex.	Yes (1)
N/A	Q5W5X9	*TTC23*	Tetratricopeptide repeat domain 23		1	ND	762	TTC23 contains tetratricopeptide repeat motifs, which serve as scaffolds to promote protein-protein interactions; however, the specific function of TTC23 is not known.	No
N/A	Q9BV40	*VAMP8*	Vesicle-associated membrane protein 8		1<br>1<br>1<br>3	209<br>215<br>215<br>257	617<br>430<br>507<br>479	VAMP8 is a soluble N-ethylmaleimide-sensitive factor attachment protein receptor (R-SNARE) involved in the coordination of autophagosome fusion with lysosome for degradation, thus maintaining cellular homeostasis.	Yes (1)
N/A	P09327	*VIL1*	Villin 1	VILLIN; VIL	1	2580	2826	VIL1 is an actin-modifying protein that plays a role in the actin nucleation, filament bundle assembly, and filament capping of calcium-regulated actin-binding proteins.	No
N/A	Q9HCK1	*ZDBF2*	Zinc finger DBF domain-containing protein 2	KIAA1571	1	1562	1855	ZDBF2 encodes a protein containing DBF4-type zinc finger domains but its specific function remains unknown.	No

The functions of the potential interaction partners were annotated according to Gene Ontology database, NCBI OMIM, and/or PubMed.

PBS, predicted biological score. A, very high confidence in the interaction; B, high confidence in the interaction; C, good confidence in the interaction; D, moderate confidence in the interaction; E, interactions involving highly connected prey domains.

N/A indicates "not applicable" and ND indicates "not determined". # represents proteins that interact with both MST3 and STK25, while ### shows proteins that interact with MST3, STK25, and MST4.

References

1. Cansby E, Kulkarni NM, Magnusson E, Kurhe Y, Amrutkar M, Nerstedt A, et al. Protein kinase MST3 modulates lipid homeostasis in hepatocytes and correlates with nonalcoholic steatohepatitis in humans. FASEB J. 2019 Sep;33(9):9974–89.
2. Khan SA, Wollaston-Hayden EE, Markowski TW, Higgins L, Mashek DG. Quantitative analysis of the murine lipid droplet-associated proteome during diet-induced hepatic steatosis. J Lipid Res. 2015 Dec;56(12):2260–72.
3. Nerstedt A, Kurhe Y, Cansby E, Caputo M, Gao L, Vorontsov E, et al. Lipid droplet-associated kinase STK25 regulates peroxisomal activity and metabolic stress response in steatotic liver. J Lipid Res. 2020 Feb;61(2):178–91.
4. Su W, Wang Y, Jia X, Wu W, Li L, Tian X, et al. Comparative proteomic study reveals 17β-HSD13 as a pathogenic protein in nonalcoholic fatty liver disease. Proc Natl Acad Sci. 2014 Aug 5;111(31):11437–42.

Supplementary Table S4. Candidate interactors of STK25 detected in a Y2H screen using STK25 as bait

PBS	Accession number	Symbol	Name	Aliases	Number of hits	Start	Stop	Function	Identified on liver lipid droplets
A	Q08379	*GOLGA2*[##]	Golgin A2	GM130	1	-38	1703	GOLGA2 is a peripheral membrane component of the *cis*-Golgi stack that maintains the Golgi apparatus structure and facilitates vesicle fusion to the Golgi membrane.	Yes (1,2)
					3	-6	883		
					4	ND	1203		
					3	ND	1241		
					1	ND	811		
					1	9	1577		
					1	9	ND		
					4	76	878		
					1	163	1224		
					11	221	846		
					4	221	1203		
					5	232	1704		
					4	233	804		
					4	242	881		
					1	259	877		
					4	343	1440		
					6	373	1030		
					2	409	1084		
					1	558	822		
					2	561	1004		
					1	736	1034		
					7	-24	1531		
					1	-4	920		
B	Q9BUL8	*PDCD10*[###]	Programmed cell death 10	CCM3; TRAF15	1	6	295	PDCD10 is a component of STRIPAK complex involved in apoptosis regulation, cell migration, and Golgi assembly.	Yes (1)
					3	33	294		
B	Q68DI1	*ZNF776*	Zinc finger protein 776		1	-37	1015	ZNF776 is a member of the Kruepple-C2H2 zinc-finger protein family with DNA and metal ion binding capacity and may be involved in the regulation of transcription; however, its specific function is unknown.	No
					2	12	1199		
					4	33	1168		
D	Q96P48	*CENTD2*	Centaurin delta-2	ARAP1; KIAA0782	1	2850	3721	ARAP1 is an ADP-ribosylation factor (ARF)-directed GTPase-activating protein (GAP) that regulates ARF-, RHO-, and CDC42-dependent cell activities.	No
D	Q9UIF9	*BAZ2A*[#]	Bromodomain adjacent to zinc finger domain protein 2A		1	2775	3091	BAZ2A is a subunit within the NoRC, that recruits the NoRC to ribosomal RNA genes, leading to their transcriptional repression.	No
D	Q9Y376	*CAB39*	Calcium-binding protein 39	MO25-α	1	51	1092	CAB39 functions as a scaffolding protein for liver kinase B1 (LKB1), facilitating the activation of the AMP-activated protein kinas (AMPK) signalling pathway and acting as a critical regulator for several STE20 kinases.	Yes (1)

PBS	Accession number	Symbol	Name	Aliases	Number of hits	Start	Stop	Function	Identified on liver lipid droplets
D	Q92820	*GGH*	Gamma-glutamyl hydrolase		1<br>1	60<br>60	ND<br>1129	GGH (EC 3.4.19.9) is a ubiquitous enzyme that hydrolyzes the γ-polyglutamate tails of pteroylpolyglutamates, converting them into the mono-glutamate form to enable their export from the cell.	No
D	O95819	*MAP4K4*[##]	Mitogen-activated protein kinase kinase kinase kinase 4	HGK; NIK	1	2094	3519	MAP4K4 (EC 2.11.1) is a serine/threonine protein kinase that belongs to the STE20 family. It plays a critical role in cell growth, signal transduction, immune function, and tumorigenesis. MAP4K4 regulates also hepatocellular lipotoxicity and NAFLD susceptibility.	No
D	Q96IZ6	*METTL2A*	Methyltransferase 2A, methylcytidine	METTL2	1	360	1310	METTL2A (EC 2.1.1) is an S-adenosyl-L-methionine-dependent methyltransferase that catalyzes the 3-methylcytidine modification at position 32 (m3C32) within the anticodon loop in mammalian $tRNA^{Thr}$, thus maintaining the stability of the tRNA architecture.	No
D	Q96G03	*PGM2*	Phosphoglucomutase 2		1	564	1823	PGM2 (EC 5.4.2.7) facilitates the intramolecular phosphate transfer on ribose or deoxyribose and plays a crucial role in the nucleoside synthesis salvage pathway.	Yes (2)
D	Q9H2K8	*TAOK3*	TAO kinase 3	JIK; DPK	2	-73	1342	TAOK3 (EC 2.7.11.1) is a serine/threonine protein kinase that belongs to the STE20 family. It is linked to MAPK signalling and is implicated in the control of immune response and tumour initiation/progression. TAOK3 also regulates hepatocellular lipotoxicity and NAFLD susceptibility.	Yes (1)
F	Q9UJ70	*NAGK*	N-acetylglucosamine kinase	GlcNAc kinase; GNK	1<br>1<br>3<br>3	15<br>201<br>246<br>348	ND<br>1082<br>1154<br>1220	NAGK (EC 2.7.1.59) converts endogenous N-acetylglucosamine (GlcNAc), a major component of complex carbohydrates, from lysosomal degradation or nutritional sources into GlcNAc 6-phosphate.	No
N/A	Q68CK6	*ACSM2B*	Acyl-CoA synthetase medium chain family, member 2B	HXMA	2	818	689	ACSM2B (EC 6.2.1.2) is a member of acyl-CoA synthetase enzymes involved in the activation of medium-chain length fatty acids and some carboxylate-containing xenobiotics.	No
N/A	Q8NBQ5	*HSD17B11*[#]	17-beta-hydroxysteroid dehydrogenase XI	17-BETA-HSDXI; RETSDR2; PAN1B	1	236	507	HSD17B11 (EC 1.1.1.62) belongs to the short-chain dehydrogenase/reductase family, involved in sex steroid, lipid, and cholesterol metabolism; it is also reported to be associated with lipid droplet formation.	Yes (1–3)
N/A	Q9NUM3	*SLC39A9*	Solute carrier family 39 (zinc transporter), member 9	ZIP9	1	2140	2745	SLC39A9 is a zinc transporter, contributing to zinc homeostasis. It also exhibits membrane androgen receptor (mAR) functionality and mediates non-classical androgen-driven actions in diverse physiological processes.	Yes (1)
N/A	Q7L7X3	*TAOK1*	TAO kinase 1	TAO1; MARKK; PSK2; KIAA1361	3	913	-224	TAOK1 (EC 2.7.11.1) is a serine/threonine protein kinase that belongs to the STE20 family. It is implicated in a range of functions including DNA damage responses, oxidative stress, apoptosis, and proliferation. TAOK1 also regulates hepatocellular lipotoxicity and NAFLD susceptibility.	Yes (1)

PBS	Accession number	Symbol	Name	Aliases	Number of hits	Start	Stop	Function	Identified on liver lipid droplets
N/A	P02766	*TTR*	Transthyretin	TBPA; PALB	1	-27	560	TTR is a tetrameric protein primarily recognized for its role in binding and transporting thyroid hormone and retinol in plasma and cerebrospinal fluid. Additionally, it may be involved in proteolysis, inflammation, and oxidative stress.	Yes (1,2)

The functions of the potential interaction partners were annotated according to Gene Ontology database, NCBI OMIM, and/or PubMed.
PBS, predicted biological score. A, very high confidence in the interaction; B, high confidence in the interaction; C, good confidence in the interaction; D, moderate confidence in the interaction.
N/A indicates "not applicable" and ND indicates "not determined". [#] represents proteins that interact with both STK25 and MST3, [##] represents proteins that interact with both STK25 and MST4, while [###] show proteins that interact with STK25, MST3, and MST4.

References

1. Cansby E, Kulkarni NM, Magnusson E, Kurhe Y, Amrutkar M, Nerstedt A, et al. Protein kinase MST3 modulates lipid homeostasis in hepatocytes and correlates with nonalcoholic steatohepatitis in humans. FASEB J. 2019 Sep;33(9):9974–89.
2. Khan SA, Wollaston-Hayden EE, Markowski TW, Higgins L, Mashek DG. Quantitative analysis of the murine lipid droplet-associated proteome during diet-induced hepatic steatosis. J Lipid Res. 2015 Dec;56(12):2260–72.
3. Crunk AE, Monks J, Murakami A, Jackman M, MacLean PS, Ladinsky M, et al. Dynamic Regulation of Hepatic Lipid Droplet Properties by Diet. Davis J, editor. PLoS ONE. 2013 Jul 11;8(7):e67631.

Supplementary Table S5. Candidate interactors of MST4 detected in a Y2H screen using MST4 as bait

PBS	Accession number	Symbol	Name	Aliases	Number of hits	Start	Stop	Function	Identified on liver lipid droplets
A	Q08379	*GOLGA2*[##]	Golgin A2	GM130	2	ND	1486	GOLGA2 is a peripheral membrane component of the *cis*-Golgi stack that maintains the Golgi apparatus structure and facilitates vesicle fusion to the Golgi membrane.	Yes (1,2)
					1	33	1308		
					2	33	ND		
					2	39	890		
					1	112	914		
					4	257	882		
					1	269	875		
					5	269	876		
					3	278	917		
					1	295	913		
					1	379	ND		
					7	409	1066		
					1	678	1475		
A	P07602	*PSAP*	Prosaposin	SAP1; SAP2	5	540	1184	PSAP is a precursor to several non-enzymatic sphingolipid activator proteins involved in sphingolipid degradation and membrane digestion. It also facilitates death receptor 6 (DR6)-induced apoptosis.	Yes (1,2)
					1	783	1217		
					1	783	1210		
					1	810	1187		
					2	822	1222		
					4	840	1196		
					1	840	1195		
					2	843	1185		
					1	936	ND		
B	P11509	*CYP2A6*	Cytochrome p450, subfamily IIA, polypeptide 6	P450PB	1	473	1369	CYP2A6 (EC 1.14.14.1) is a member of the cytochrome p450 superfamily of monooxygenases that encodes a functional phase I enzyme. It is involved in the hydroxylation of anti-cancer drugs, metabolic activation of procarcinogens, and the biotransformation of various endogenous compounds.	No
					1	1073	1676		
B	Q9BUL8	*PDCD10*[###]	Programmed cell death 10	CCM3; TRAF15	1	25	587	PDCD10 is a component of STRIPAK complex involved in apoptosis regulation, cell migration, and Golgi assembly.	Yes (1)
					2	45	ND		
					1	45	144		
D	A8MT69	*CENPX*	Centromeric protein X	STRA13; FAAP10; MHF2; D9	1	114	519	CENPX is a component of the fanconi anemia (FA) core complex which serves as a co-factor of FA complement group M (FANCM) required for cellular resistance to DNA crosslinking agents, contributing to genomic stability.	No
D	P10909	*CLU*	Clusterin	SGP2; APOJ; CLI; TRPM2	1	1203	1514	CLU is a chaperone protein that scavenges and clears cellular debris and misfolded proteins, and also regulates apoptosis.	Yes (1,2)
D	O00303	*EIF3F*	Eukaryotic translation initiation factor 3, subunit F		1	57	237	EIF3F (EC 3.4.19.12) is a subunit of the eukaryotic initiation factor eIF3 required for the initiation of protein synthesis. EIF3F negatively regulates cell growth and induces apoptosis.	Yes (1,2)

PBS	Accession number	Symbol	Name	Aliases	Number of hits	Start	Stop	Function	Identified on liver lipid droplets
D	P10321	*HLA-Cw*17*	HLA class I histocompatibility antigen, C alpha chain 17		1	381	720	HLA-Cw*17 is a variant of the HLA-C gene, playing a crucial role in antigen presentation, immune response regulation, and transplantation compatibility.	No
D	P24001	*IL32*	Interleukin 32	NK4	1	231	681	IL32 is an intracellular cytokine that acts as a switch between pro-inflammatory and anti-inflammation responses to mediate tumorigenesis and metabolic processes. It has been reported to associate with lipid droplet membranes and vascular structures.	No
D	O95819	*MAP4K4*##	Mitogen-activated protein kinase kinase kinase kinase 4	HGK; NIK	1	2190	3515	MAP4K4 (EC 2.11.1) is a serine/threonine protein kinase that belongs to the STE20 family. It plays a critical role in cell growth, signal transduction, immune function, and tumorigenesis. MAP4K4 also regulates hepatocellular lipotoxicity and NAFLD susceptibility.	No
D	Q9Y478	*PRKAB1*	Protein kinase AMP-activated non-catalytic subunit beta 1	AMPK-beta-1	1	603	1562	PRKAB1 is a non-catalytic subunit of AMP-activated protein kinase (AMPK), an energy sensor protein kinase that plays a key role in regulating cellular energy metabolism.	No
D	Q9UKE5	*TNIK*	TRAF2 and NCK-interacting kinase	KIAA0551	1	2685	4059	TNIK (EC 2.7.11.1) is a serine/threonine kinase that interacts with Ras-Related Protein Rap-2a (Rap2) to regulate actin cytoskeleton and activates Wnt/β-catenin and JNK pathways.	No
E	Q05516	*ZBTB16*	Zinc finger and BTB domain-containing protein 16	ZNF145; PLZF	1	1143	2304	ZBTB16 belongs to the Zinc finger and Broad-Complex, Tramtrack, and Bric-a-Brac (BTB) domain-containing transcription factor family. Its BTB domain controls the transcriptional activity of ZBTB16 involved in chromatin remodelling and other cellular processes. Additionally, ZBTB16 is a recognised pleiotropic mediator of metabolic disorders.	No
N/A	P12694	*BCKDHA*	Branched-chain keto acid dehydrogenase E1, alpha polypeptide	BCKDE1A	1	780	149	BCKDHA (EC 1.2.4.4) is the catalytic subunit of mitochondrial branched-chain alpha-ketoacid dehydrogenase (BCKD) that converts branched-chain keto acids (BCKA) into acyl-CoA via oxidative decarboxylation, ultimately contributing to energy production.	Yes (1)
N/A	Q9Y5B0	*CTDP1*	C-terminal domain of RNA polymerase II subunit A, phosphatase of, subunit 1	FCP1	1	761	3040	CTDP1 (EC 3.1.3.16) is a member of the halocid dehalogenase superfamily phosphatases and plays a critical role in transcription via dephosphorylation of the C-terminal domain of DNA-directed RNA polymerase II. It is also crucial in the regulation of exit from mitosis and DNA damage response.	No
N/A	P07339	*CTSD*	Cathepsin D		1	1799	1375	CTSD (EC 3.4.23.5) encodes Cathepsin D, a lysosomal protease, with key roles in protein breakdown, autophagy, neural function, and apoptosis.	Yes (1,2)
N/A	Q14999	*CUL7*	Cullin 7	KIAA0076	1	ND	2275	The CUL7 is a member of the DOC domain-containing cullin family and forms part of the core components of ubiquitin ligase complex. It is involved in protein degradation, cell growth, cell cycle regulation, and DNA repair, with implications for various malignant tumours.	No

The functions of the potential interaction partners were annotated according to Gene Ontology database, NCBI OMIM, and/or PubMed.
PBS, predicted biological score. A, very high confidence in the interaction; B, high confidence in the interaction; C, good confidence in the interaction; D, moderate confidence in the interaction; E, interactions involving highly connected prey domains.
N/A indicates "not applicable" and ND indicates "not determined". ## represents proteins that interact with both MST4 and STK25, while ### shows proteins that interact with MST4, MST3, and STK25.

References

1. Cansby E, Kulkarni NM, Magnusson E, Kurhe Y, Amrutkar M, Nerstedt A, et al. Protein kinase MST3 modulates lipid homeostasis in hepatocytes and correlates with nonalcoholic steatohepatitis in humans. FASEB J. 019 Sep;33(9):9974–89.
2. Khan SA, Wollaston-Hayden EE, Markowski TW, Higgins L, Mashek DG. Quantitative analysis of the murine lipid droplet-associated proteome during diet-induced hepatic steatosis. J Lipid Res. 015 Dec;56(12):2260–72.

Supplementary Table S6. Abundance of molecular lipid species in IHHs transfected with *MST3*, *STK25*, *MST4*, *HSD17B11*, and/or NTC siRNA

**Lipid species**	**Means ± SEM**				***P* value *versus* control**		
	**Control**	**HSD17B11 KD**	**tKD**	**HSD17B11 KD +tKD**	**HSD17B11 KD**	**tKD**	**HSD17B11 KD +tKD**
**TAG 48:0**	11.8±1.3	9.7±0.1	8.5±1.3	11.4±0.8	0.23	0.10	0.82
**TAG 48:1**	15.3±1.3	12.4±0.5	12.6±0.5	14.1±0.6	0.06	0.07	0.43
**TAG 48:2**	4.6±0.3	4.2±0.1	4.6±0.08	4.6±0.2	0.27	0.87	0.95
**TAG 48:3**	0.4±0.03	0.4±0.03	0.5±0.01	0.5±0.02	0.58	**0.004**	0.10
**TAG 50:0**	1.2±0.2	1.2±0.1	0.8±0.1	1.4±0.1	0.99	0.06	0.56
**TAG 50:1**	30.1±3.2	25.0±1.2	21.3±1.2	28.0±1.3	0.16	**0.02**	0.55
**TAG 50:2**	18.4±1.6	16.6±0.6	15.4±0.4	17.6±0.7	0.32	0.09	0.68
**TAG 50:3**	2.6±0.2	2.6±0.09	2.7±0.05	2.8±0.1	0.97	0.44	0.35
**TAG 50:4**	0.05±0.005	0.05±0.003	0.06±0.001	0.06±0.002	0.76	0.09	0.66
**TAG 52:0**	0.1±0.02	0.1±0.01	0.06±0.009	0.1±0.01	0.56	0.10	0.50
**TAG 52:1**	3.5±0.4	3.2±0.1	2.0±0.1	3.3±0.2	0.47	**0.004**	0.77
**TAG 52:2**	22.7±2.1	21.2±0.6	15.7±0.4	21.2±0.8	0.52	**0.006**	0.53
**TAG 52:3**	6.0±0.5	6.3±0.2	5.4±0.1	6.4±0.2	0.63	0.27	0.50
**TAG 52:4**	0.4±0.03	0.4±0.01	0.4±0.01	0.4±0.02	0.64	0.75	0.77
**TAG 52:5**	0.04±0.004	0.03±0.001	0.04±0.0009	0.04±0.003	0.27	0.89	0.93
**TAG 54:1**	0.2±0.02	0.2±0.01	0.1±0.008	0.2±0.01	0.68	**0.03**	0.49
**TAG 54:2**	2.1±0.2	2.1±0.08	1.3±0.04	2.0±0.08	0.88	**0.004**	0.87
**TAG 54:3**	4.0±0.3	5.1±0.2	3.3±0.09	4.9±0.2	**0.009**	0.07	**0.03**
**TAG 54:4**	0.5±0.04	0.5±0.01	0.4±0.01	0.5±0.02	0.65	0.17	0.43
**TAG 54:5**	0.2±0.03	0.2±0.005	0.2±0.005	0.2±0.008	0.08	0.13	0.25
**TAG 54:6**	0.4±0.04	0.3±0.007	0.3±0.01	0.3±0.01	**0.002**	0.06	**0.02**
**TAG 54:7**	0.1±0.01	0.09±0.003	0.1±0.004	0.1±0.004	**0.002**	0.67	**0.03**
**TAG 54:8**	0.02±0.01	0.01±0.0009	0.02±0.0007	0.01±0.0004	**0.004**	0.10	**0.01**
**TAG 56:1**	0.05±0.006	0.04±0.003	0.04±0.0004	0.05±0.004	0.41	0.14	0.49
**TAG 56:2**	0.1±0.01	0.1±0.006	0.08±0.004	0.1±0.006	0.76	**0.03**	0.90
**TAG 56:3**	0.4±0.03	0.5±0.02	0.3±0.008	0.4±0.02	**0.02**	**0.03**	0.07
**TAG 56:4**	0.06±0.005	0.07±0.003	0.05±0.001	0.07±0.003	0.05	**0.049**	0.07
**TAG 56:5**	0.1±0.01	0.1±0.004	0.09±0.003	0.1±0.006	0.12	**0.01**	0.50
**TAG 56:6**	0.2±0.02	0.1±0.007	0.1±0.007	0.1±0.01	0.13	0.10	0.37
**TAG 56:7**	0.4±0.03	0.2±0.007	0.3±0.008	0.3±0.01	**0.002**	**0.04**	**0.01**
**TAG 56:8**	0.07±0.006	0.05±0.003	0.07±0.002	0.06±0.002	**0.007**	0.76	0.07
**TAG 58:2**	0.06±0.007	0.05±0.003	0.04±0.002	0.06±0.003	0.42	0.09	0.91
**TAG 58:3**	0.05±0.005	0.06±0.003	0.04±0.001	0.06±0.003	0.07	0.20	0.15
**TAG 58:6**	0.04±0.005	0.04±0.002	0.03±0.001	0.04±0.001	0.71	**0.03**	0.78
**TAG 58:7**	0.04±0.004	0.03±0.002	0.03±0.001	0.04±0.002	0.41	0.10	0.57
**TAG 58:8**	0.08±0.008	0.07±0.003	0.08±0.002	0.08±0.003	0.25	0.55	0.77
**Cer 14:0**	5.5±0.2	4.9±0.3	4.7±0.1	4.7±0.2	0.13	**0.01**	**0.01**
**Cer 16:0**	335.5±16.1	314.8±15.5	261.5±7.2	293.6±14.0	0.37	**0.0009**	0.07
**Cer 18:0**	28.7±1.6	28.2±1.5	21.6±0.6	24.5±1.1	0.81	**0.001**	0.05
**Cer 20:0**	12.8±0.8	15.3±0.8	9.0±0.3	12.4±0.56	**0.045**	**0.0004**	0.72
**Cer 22:0**	53.8±3.1	59.0±3.0	38.0±1.1	50.7±2.3	0.24	**0.0003**	0.44
**Cer 24:0**	100.8±5.3	100.3±4.5	83.1±2.3	92.8±3.8	0.93	**0.008**	0.23
**Cer 24:1**	200.9±9.8	204.0±8.5	138.2±3.4	181.6±7.6	0.82	**0.00003**	0.14

IHHs were transfected with different combinations of *MST3*, *STK25*, *MST4*, *HSD17B11*, and/or NTC siRNA, and incubated with oleic and palmitic acid for 48 h post-transfection. TAG and ceramide species are expressed in nmol/mg protein and pmol/mg protein, respectively. Data are mean ± SEM from 7-8 cell culture wells per group. Cer, ceramide; KD, knockdown; tKD, triple knockdown. Bold text indicates significant *P* values.

Supplementary Table S7. Candidate substrates of MST3, STK25, and/or MST4 detected in an integrative analysis of substrate screen data with genome-scale metabolic model Human1

Accession no.	Symbol	Name	Aliases	Phosphory-lation site	Function	Subcellular localization	Detected in screen for
O95477	*ABCA1*	ATP-binding cassette subfamily a member 1	ABC1; CERP	s1255*	ABCA1 (EC 7.6.2.1) is a transmembrane protein that belongs to the ATP-binding cassette superfamily and catalyzes the translocation of specific phospholipids, participating in phospholipid transfer to apolipoproteins to form nascent high-density lipoproteins. Hepatic ABCA1 protein levels are negatively correlated with human MASH and liver fibrosis (1).	ER, plasma membrane, LD	STK25 MST4
P08183	*ABCB1*	ATP-binding cassette subfamily b member 1	PGY1; MDR1; GP170	s683*	ABCB1 (EC 3.6.3.44) is a transmembrane protein that belongs to the ATP-binding cassette superfamily and functions to translocate drugs and phospholipids across the plasma membrane. Hepatic *ABCB1* mRNA expression is increased in MASH patients compared with healthy controls (2).	Plasma membrane, extracellular	MST3 MST4
P09917	*ALOX5*	Arachidonate 5-lipoxygenase	LOG5; 5-LO	s272*	ALOX5 (EC 1.13.11.34) catalyzes the oxygenation of arachidonate to 5-hydroperoxyeicosatetraenoate followed by its dehydration to 5,6-epoxyeicosatetraenoate, thus performing the first two steps in the biosynthesis of leukotrienes, which are potent mediators of inflammation. Hyperlipidemic mice lacking ALOX5 are protected against high-fat diet-induced liver injury and meta-inflammation and hepatic ALOX5 protein abundance is increased in patients with MASH and liver fibrosis compared with healthy controls (3, 4).	Cytosol, LD	MST3 STK25 MST4
Q92793	*CREBBP*	CREB-binding protein	CBP	s437*	CREBBP (EC 2.3.1.48) regulates transcription by acetylating histones and thereby tagging them for transcriptional activation. *Crebbp* knockout mice challenged with a high-fat diet develop more severe MASH compared with wild-type controls (5).	Nucleus, cytosol	MST3 MST4
Q16760	*DGKD*	Diacylglycerol kinase delta 130-kDa	KIAA0145	s66*	DGKD (EC 2.7.1.107) phosphorylates diacylglycerol to produce phosphatidic acid, which both act as second messengers in various signaling cascades. However, the possible role of DGKD in MASLD/MASH development is not known.	Nucleus, cytosol, plasma membrane	MST4
P00533	*EGFR*	Epidermal growth factor receptor	ERBB1; HER1; SA7	s768*	EGFR (EC 2.7.10.1) is a receptor tyrosine kinase binding ligands of the EGF family and activating diverse signaling cascades to convert extracellular cues into appropriate cellular responses. Pharmacological inhibition of EGFR in high-fat diet-fed mice prevents the development of hepatic steatosis, fibrosis, and oxidative stress, and EGFR antagonists have been proposed as a treatment strategy for MASLD (6, 7).	Plasma membrane, extracellular, LD	STK25 MST4
Q06210	*GFPT1*	Glutamine:fructose-6-phosphate amidotransferase 1	GFAT1; GFAT	s235*	GFPT1 (EC 2.6.1.16) catalyzes the formation of glucosamine 6-phosphate from fructose 6-phosphate, the first step in the hexosamine biosynthesis pathway. GFPT1 is linked to lipid accumulation, ER stress, and inflammatory pathway activation in human hepatocytes *in vitro* and hepatic *Gfpt1* mRNA expression is increased in methionine and choline deficient diet-fed mice (8, 9).	Cytosol, LD	MST3 STK25 MST4
O94808	*GFPT2*	Glutamine:fructose-6-phosphate amidotransferase 2	GFAT2	s202*	GFPT2 (EC 2.6.1.16) is a paralog of GFPT1 and catalyzes the formation of glucosamine 6-phosphate from fructose 6-phosphate. However, the possible role of GFPT2 in MASLD/MASH development is not known.	Cytosol	MST3 MST4
P25098	*GRK2*	G protein-coupled receptor kinase 2	ADRBK1; BARK1	s685*	GRK2 (EC 2.7.11) phosphorylates the agonist-occupied form of the beta-adrenergic and closely related receptors and is also involved in lipolysis by inhibiting insulin signaling. *Grk2*$^{+/-}$ mice are protected against high-fat diet-induced MASLD development compared with wild-type controls and GRK2 mRNA and protein levels are positively correlated with the severity of MASH in humans (10).	Plasma membrane, cytosol	STK25 MST4

Accession no.	Symbol	Name	Aliases	Phosphory-lation site	Function	Subcellular localization	Detected in screen for
Q00987	*MDM2*	MDM2 protooncogene	HDM2	s166*	MDM2 (EC 2.3.2.27) is a E3 ubiquitin-protein ligase that mediates ubiquitination of p53/TP53, leading to its degradation by the proteasome. MDM2 acts as a negative regulator of hepatic VLDL secretion and MDM2 protein levels in the liver are positively correlated with the severity of MASH in humans as well as in mouse models of diet-induced MASH (11).	Nucleus, cytosol, plasma membrane	MST3 STK25 MST4
Q16875	*PFKFB3*	6-phosphofructo-2-kinase/fructose-2,6-bisphosphatase 3	PFKFB; IPFK2	s461*	PFKFB3 (EC 2.7.1.105) catalyzes both the synthesis and degradation of fructose 2,6-bisphosphate during glycolysis. Myeloid cell-specific genetic disruption of PFKFB3 exacerbates the severity of high-fat diet-induced hepatic steatosis and inflammation in mice (12).	Cytosol	MST3 STK25 MST4
P47712	*PLA2G4A*	Phospholipase A2	CPLA2-alpha	s515*	PLA2G4A (EC 3.1.1.5) catalyzes the hydrolysis of membrane phospholipids to release arachidonic acid, which is subsequently metabolized into eicosanoids. Eicosanoids are lipid-based hormones that regulate a wide range of cellular functions such as cell growth, hemodynamics, and inflammatory responses. However, the possible role of PLA2G4A in MASLD/MASH development is not known.	Golgi apparatus, cytosol, LD	STK25
Q9NQ66	*PLCB1*	Phospholipase C beta 1	KIAA0581	s887*	PLCB1 (EC 3.1.4.11) catalyzes the generation of inositol 1,4,5-trisphosphate (IP3) and diacylglycerol from phosphatidylinositol 4,5-bisphosphate (IP2), a key step in the intracellular transduction of many extracellular signals. However, the possible role of this PLCB1 in MASLD/MASH development is not known.	Cytosol, nucleus	MST4
Q15139	*PRKD1*	Protein kinase D1	PRKCM; PKD	s738*	PRKD1 (EC 2.7.11.13) is a serine/threonine protein kinase that phosphorylates several different targets including EGFR and NLRP3, and thereby regulates a variety of cellular functions such as cell proliferation and inflammatory activation. *Prkd1* knockout mice fed a high-fat diet are protected against liver steatosis and MASLD development (13).	Golgi apparatus, cytosol, plasma membrane	MST3 STK25 MST4
O60260	*PRKN*	Parkin RBR E3 ubiquitin protein ligase	parkin; PARK2	s101*	PRKN (EC 2.3.2.31) is a RING domain-containing E3 ubiquitin ligase involved in proteasome-dependent degradation of proteins. PRKN is also important for mitochondrial quality control by lysosome-dependent degradation of damaged mitochondria through mitophagy. Liver-specific loss of PRKN accelerates the onset of MASLD in western type diet-fed mice (14).	Nucleus, cytosol, mitochondria	STK25 MST4
P21673	*SAT1*	Spermidine/spermine n(1)-acetyltransferase 1	SSAT; SSAT1	t10	SAT1 (EC 2.3.1.57) catalyzes the acetylation of the polyamines spermidine and spermine, resulting in their catabolism. Pharmacological activation of SAT1 in high-fat-fed mice results in reduced hepatic steatosis (15).	Cytosol	MST3
P11168	*SLC2A2*	Solute carrier family 2, facilitated glucose transporter, member 2	GLUT2	s491*	SLC2A2 is a facilitative hexose transporter that mediates the transport of glucose, fructose, and galactose through the plasma membrane. The cellular uptake of fructose in specific has been linked to MASLD development and liver-specific *Slc2a2* knockout mice demonstrate reduced hepatic lipid levels compared with wild-type controls (16).	Plasma membrane, LD	MST3 STK25 MST4
P19634	*SLC9A1*	Solute carrier family 9, member 1	APNH; NHE1	s703*	SLC9A1 is involved in pH regulation to eliminate acids generated by active metabolism. SLC9A1-deficient mice display reduced hepatic lipogenesis, fibrosis, and cellular stress (17).	Plasma membrane, ER membrane	MST3 STK25 MST4
P52888	*THOP1*	Thimet oligopeptidase 1	TOP	s643*	THOP1 (EC 3.4.24.15) is an oligopeptidase that cleaves cytosolic peptides, preferentially bonds with hydrophobic residues. Although high-fat diet-fed *Thop1* knockout mice display less liver steatosis compared with wild-type controls, human *THOP1* mRNA expression in the liver is negatively correlated with MASH severity (18, 19).	Nucleus, cytosol	STK25 MST4

Accession no.	Symbol	Name	Aliases	Phosphory-lation site	Function	Subcellular localization	Detected in screen for
P22314	*UBA1*	Ubiquitin-like modifier-activating enzyme 1	UBE1; GXP1	S835*	UBA1 (EC 6.2.1.45) catalyzes the first step in ubiquitin conjugation to mark cellular proteins for degradation through the ubiquitin-proteasome system. However, the possible role of UBA1 in MASLD/MASH development is not known.	Nucleus, cytosol, mitochondria, LD	STK25

The functions and subcellular localizations of the potential targets were annotated according to Gene Ontology database, NCBI OMIM, GeneCards, and/or PubMed.
*Phosphosites annotated in PhosphoSitePlus
LD, lipid droplet

References

1. Vega-Badillo J, Gutierrez-Vidal R, Hernandez-Perez HA, Villamil-Ramirez H, Leon-Mimila P, Sanchez-Munoz F, Moran-Ramos S, et al. Hepatic miR-33a/miR-144 and their target gene ABCA1 are associated with steatohepatitis in morbidly obese subjects. Liver Int 2016;36:1383-1391.
2. Hardwick RN, Fisher CD, Canet MJ, Scheffer GL, Cherrington NJ. Variations in ATP-binding cassette transporter regulation during the progression of human nonalcoholic fatty liver disease. Drug Metab Dispos 2011;39:2395-2402.
3. Martinez-Clemente M, Ferre N, Gonzalez-Periz A, Lopez-Parra M, Horrillo R, Titos E, Moran-Salvador E, et al. 5-lipoxygenase deficiency reduces hepatic inflammation and tumor necrosis factor alpha-induced hepatocyte damage in hyperlipidemia-prone ApoE-null mice. Hepatology 2010;51:817-827.
4. Pu S, Li Y, Liu Q, Zhang X, Chen L, Li R, Zhang J, et al. Inhibition of 5-Lipoxygenase in Hepatic Stellate Cells Alleviates Liver Fibrosis. Front Pharmacol 2021;12:628583.
5. Zhang C, Wang G, Zheng Z, Maddipati KR, Zhang X, Dyson G, Williams P, et al. Endoplasmic reticulum-tethered transcription factor cAMP responsive element-binding protein, hepatocyte specific, regulates hepatic lipogenesis, fatty acid oxidation, and lipolysis upon metabolic stress in mice. Hepatology 2012;55:1070-1082.
6. Bhushan B, Banerjee S, Paranjpe S, Koral K, Mars WM, Stoops JW, Orr A, et al. Pharmacologic Inhibition of Epidermal Growth Factor Receptor Suppresses Nonalcoholic Fatty Liver Disease in a Murine Fast-Food Diet Model. Hepatology 2019;70:1546-1563.
7. Liang D, Chen H, Zhao L, Zhang W, Hu J, Liu Z, Zhong P, et al. Inhibition of EGFR attenuates fibrosis and stellate cell activation in diet-induced model of nonalcoholic fatty liver disease. Biochim Biophys Acta Mol Basis Dis 2018;1864:133-142.
8. Sage AT, Walter LA, Shi Y, Khan MI, Kaneto H, Capretta A, Werstuck GH. Hexosamine biosynthesis pathway flux promotes endoplasmic reticulum stress, lipid accumulation, and inflammatory gene expression in hepatic cells. Am J Physiol Endocrinol Metab 2010;298:E499-511.
9. Lee DE, Lee SJ, Kim SJ, Lee HS, Kwon OS. Curcumin Ameliorates Nonalcoholic Fatty Liver Disease through Inhibition of O-GlcNAcylation. Nutrients 2019;11.
10. Cruces-Sande M, Vila-Bedmar R, Arcones AC, Gonzalez-Rodriguez A, Rada P, Gutierrez-de-Juan V, Vargas-Castrillon J, et al. Involvement of G protein-coupled receptor kinase 2 (GRK2) in the development of non-alcoholic steatosis and steatohepatitis in mice and humans. Biochim Biophys Acta Mol Basis Dis 2018;1864:3655-3667.
11. Lin H, Wang L, Liu Z, Long K, Kong M, Ye D, Chen X, et al. Hepatic MDM2 Causes Metabolic Associated Fatty Liver Disease by Blocking Triglyceride-VLDL Secretion via ApoB Degradation. Adv Sci (Weinh) 2022;9:e2200742.
12. Ma L, Li H, Hu J, Zheng J, Zhou J, Botchlett R, Matthews D, et al. Indole Alleviates Diet-Induced Hepatic Steatosis and Inflammation in a Manner Involving Myeloid Cell 6-Phosphofructo-2-Kinase/Fructose-2,6-Biphosphatase 3. Hepatology 2020;72:1191-1203.
13. Loffler MC, Mayer AE, Trujillo Viera J, Loza Valdes A, El-Merahbi R, Ade CP, Karwen T, et al. Protein kinase D1 deletion in adipocytes enhances energy dissipation and protects against adiposity. EMBO J 2018;37.
14. Undamatla R, Fagunloye OG, Chen J, Edmunds LR, Murali A, Mills A, Xie B, et al. Reduced mitophagy is an early feature of NAFLD and liver-specific PARKIN knockout hastens the onset of steatosis, inflammation and fibrosis. Sci Rep 2023;13:7575.
15. Castoldi F, Hyvonen MT, Durand S, Aprahamian F, Sauvat A, Malik SA, Baracco EE, et al. Chemical activation of SAT1 corrects diet-induced metabolic syndrome. Cell Death Differ 2020;27:2904-2920.
16. Seyer P, Vallois D, Poitry-Yamate C, Schutz F, Metref S, Tarussio D, Maechler P, et al. Hepatic glucose sensing is required to preserve beta cell glucose competence. J Clin Invest 2013;123:1662-1676.
17. Prasad V, Chirra S, Kohli R, Shull GE. NHE1 deficiency in liver: implications for non-alcoholic fatty liver disease. Biochem Biophys Res Commun 2014;450:1027-1031.
18. Gewehr MCF, Teixeira AAS, Santos BAC, Biondo LA, Gozzo FC, Cordibello AM, Eichler RAS, et al. The Relevance of Thimet Oligopeptidase in the Regulation of Energy Metabolism and Diet-Induced Obesity. Biomolecules 2020;10.
19. Zhu Y, Zhang H, Jiang P, Xie C, Luo Y, Chen J. Transcriptional and Epigenetic Alterations in the Progression of Non-Alcoholic Fatty Liver Disease and Biomarkers Helping to Diagnose Non-Alcoholic Steatohepatitis. Biomedicines 2023;11.
